# A Novel Subfamily GH13_46 of the α-Amylase Family GH13 Represented by the Cyclomaltodextrinase from *Flavobacterium* sp. No. 92

**DOI:** 10.3390/molecules27248735

**Published:** 2022-12-09

**Authors:** Filip Mareček, Štefan Janeček

**Affiliations:** 1 Laboratory of Protein Evolution, Institute of Molecular Biology, Slovak Academy of Sciences, SK-84551 Bratislava, Slovakia; 2 Department of Biology, Faculty of Natural Sciences, University of SS. Cyril and Methodius, SK-91701 Trnava, Slovakia

**Keywords:** α-amylase family GH13, GH13 subfamilies, cyclomaltodextrinase, in silico analysis, conserved sequence regions, evolutionary relationships

## Abstract

In the CAZy database, the α-amylase family GH13 has already been divided into 45 subfamilies, with additional subfamilies still emerging. The presented in silico study was undertaken in an effort to propose a novel GH13 subfamily represented by the experimentally characterized cyclomaltodxtrinase from *Flavobacterium* sp. No. 92. Although most cyclomaltodextrinases have been classified in the subfamily GH13_20. This one has not been assigned any GH13 subfamily as yet. It possesses a non-specified immunoglobulin-like domain at its N-terminus mimicking a starch-binding domain (SBD) and the segment MPDLN in its fifth conserved sequence region (CSR) typical, however, for the subfamily GH13_36. The searches through sequence databases resulted in collecting a group of 108 homologs forming a convincing cluster in the evolutionary tree, well separated from all remaining GH13 subfamilies. The members of the newly proposed subfamily share a few exclusive sequence features, such as the “aromatic” end of the CSR-II consisting of two well-conserved tyrosines with either glycine, serine, or proline in the middle or a glutamic acid succeeding the catalytic proton donor in the CSR-III. Concerning the domain N of the representative cyclomaltodextrinase, docking trials with α-, β- and γ-cyclodextrins have indicated it may represent a new type of SBD. This new GH13 subfamily has been assigned the number GH13_46.

## 1. Introduction

Within the CAZy database sequence-based classification of glycoside hydrolases (GHs) [1], the family GH13, also known as the main α-amylase family [2], represents the original, largest and most deeply studied GH family with the α-amylase specificity [3]. From the beginning, i.e., from the 90s of the previous century, the α-amylase family GH13 has been established as a polyspecific family of several various amylolytic enzymes, such as α-amylase, cyclodextrin glucanotransferase, α-glucosidase, pullulanase, and others [4,5,6]. Currently, the GH13 scope is enormous—with more than 138 thousand members [1], the family covers around 30 different enzyme specificities from hydrolases (EC 3), transferases (EC 2), and isomerases (EC 5), the non-enzymatic heavy-chains of heterodimeric transport proteins (rBAT and 4F2hc) being also involved [2,3,4,5,6,7].

The family GH13 can briefly be characterized by a few basic criteria as follows [2,8,9,10,11,12,13]: (i) its members adopt the fold of a (β/α)_8_-barrel (TIM-barrel) as a catalytic domain employing the retaining reaction mechanism; (ii) the catalytic machinery consists of a catalytic nucleophile (aspartic acid), a proton donor (glutamic acid) and a transition-state stabilizer (aspartic acid) at the strands β4, β5 and β7, respectively; and (iii) sequences share from 4 up to 7 typical conserved sequence regions (CSRs). The canonical domain organization in the family GH13 consists of three domains: A (catalytic TIM-barrel), B (it protrudes out of the barrel in the place of the loop 3 connecting the strand β3 with the helix α3), and C (succeeding the catalytic TIM-barrel), although various mainly starch-binding domains (SBDs) classified as a different carbohydrate-binding module (CBM) families being also found [1,12,14].

At the higher level of the CAZy hierarchy, the family GH13 has been grouped with related families GH70 and GH77 into the clan GH-H [15], whereas, at the lower hierarchy level, it has been divided into GH13 subfamilies [16]. Originally, the CAZy curators divided the family into 35 GH13 subfamilies in 2006 [16], but until now, 45 official GH13 subfamilies in total have been established [1]. This is a continuous process that also reflects recommendations in the published literature, such as e.g., GH13_43 [17], GH13_44 [18], and GH13_45 [19,20,21]. In the database itself, some further GH13 members or groups of sequences may still await their official recognition in CAZy [1]. It is worth mentioning, however, that two subfamilies—the so-called oligo-1,6-glucosidase and neopullulanase subfamilies—have been proposed before the official division of the family GH13 occurred [22]. That proposal was based on a specific sequence feature present in the fifth CSR of those GH13 members, the signature being either QPDLN for the oligo-1,6-glucosidase subfamily or MPKLN for the neopullulanase subfamily. Currently, these two “unofficial” subfamilies cover several CAZy-official GH13 subfamilies: (i) 4, 16, 17, 18, 23, 29, 30, 31, 34, and 35; and (ii) 20 and 21 [1] with specificities such as oligo-1,6-glucosidase, α-glucosidase, dextran glucosidase, trehalose-6-phosphate hydrolase, amylosucrase, sucrose phosphorylase, isomaltulose synthase and trehalose synthase for the former subfamily, whereas neopullulanase, cyclomaltodextrinase and maltogenic amylase for the latter one [22]. That study identified even an intermediary group of amylolytic enzymes exhibiting a mixed enzyme specificity of α-amylase, cyclomaltodextrinase, and neopullulanase with the sequence signature MPDLN in the CSR-V, which has later been assigned the subfamily GH13_36 [23].

The present study has been undertaken in an effort to emphasize the relevancy of creating a novel GH13 subfamily around the cyclomaltodextrinase from *Flavobacterium* sp. No. 92, whose three-dimensional structure was published already in 2003 [24]. The enzyme itself has identified almost 20 years ago [25] and subsequently characterized as rather a powerful decycling maltodextrinase degrading starch and pullulan, being able to perform also transglycosylations [26,27,28]. Despite its quite complex structure/function characterization [29], this cyclomaltodextrinase has not been assigned any GH13 subfamily until now [1]. It really may represent a unique amylolytic enzyme with regard to what has already been known for typical members of the neopullulanase subfamily [30]. The reasons for that are double: (i) it contains a CBM-like domain at its N-terminus—a feature similar to that of typical neopullulanases (cyclomaltodextrinases) having the N-terminal SBD of the family CBM34 [14,31,32,33,34]; and, more remarkably, (ii) it possesses the sequence MPDLN in its fifth CSR—a signature of the so-called intermediary group of amylolytic enzymes classified already in the subfamily GH13_36 [22,23]. Note that typical neopullulanases (subfamilies GH13_20 and GH13_21) usually have the CSR-V with the lysine in the middle of the region (instead of an aspartic acid characteristic for the members of the oligo-1,6-glucosidase subfamily [22]. Finally, it also should be pointed out that there are at least three experimentally characterized family GH13 members, i.e., a neopullulanase SusA from *Bacteroides thetaiotaomicron* [35], an α-amylase AmyZ from *Zunongwangia profunda* [36] and a cyclomaltodextrinase from *Massilia timonae* [37], which share with the *Flavobacterium* sp. No. 92 cyclomaltodextrinase, all the particular features of interest mentioned above.

All these attributes make thus the cyclomaltodextrinase from *Flavobacterium* sp. No. 92 is an attractive subject worth deep in silico studies that should be helpful in elucidating its position within the entire α-amylase family GH13. Therefore, the aim of the present study has been to deliver the comprehensive results from such a bioinformatics analysis convincing enough in order to define a novel GH13 subfamily, the subfamily GH13_46, represented just by this unique cyclomaltodextrinase.

## 2. Materials and Methods

### 2.1. Sequence Collection

The cyclomaltodextrinase from *Flavobacterium* sp. No. 92 [24], neopullulanase SusA from *Bacteroides thetaiotaomicron* [35], α-amylase AmyZ from *Zunongwangia profunda* [36], and cyclomaltodextrinase from *Massilia timonae* [37] were selected as the main representatives of the potentially new GH13 subfamily. As yet, none of the four has been assigned to any GH13 subfamily within the CAZy database ([1]; http://www.cazy.org/, accessed on 29 October 2022), and they all share two specific features: (i) the intermediary character of the sequence in the fifth CSR—MPDxN [22,23]; and (ii) the presence of a CBM-like domain at their N-termini currently not classified as a CBM family [14,30]. Similar hypothetical enzymes from the family GH13 have been obtained by the protein BLAST search ([38]; https://blast.ncbi.nlm.nih.gov/, accessed on 1 October 2020), using the amino acid sequence of the *Flavobacterium* cyclomaltodextrinase (UniProt Accession No.: Q8KKG0) as a query. In total, three searches with the same cyclomaltodextrinase query sequence were performed, i.e., separately limiting the searched databases to kingdoms of *Bacteria*, *Archaea* and *Eucarya*. With regard to sources of sequences caught by BLAST, one non-redundant amino acid sequence was selected to represent each species and/or bacterial strain. Furthermore, the simultaneous presence of three sequence-structural features was considered as basic criterium for sequence selection: (i) the N-terminal module homologous to that present in the cyclomaltodextrinase from *Flavobacterium* sp. No. 92 [24]; (ii) up to seven CSRs established for the α-amylase family GH13 [10] with a special emphasis on the CSR-V [22,23]; and (iii) intact catalytic machinery of the family GH13 [2], i.e., the catalytic triad of aspartic acid, glutamic acid and aspartic acid acting as a catalytic nucleophile (strand β4; CSR-II), proton donor (strand β5; CSR-III) and transition-state stabilizer (strand β7, CSR-IV), respectively. By the above-mentioned procedure, the set of 108 (including the query) studied sequences was obtained (Table S1).

In order to perform a convincing comparison covering the entire α-amylase family GH13, the selected set of the sequences forming the potential novel GH13 subfamily has been further completed by 145 sequences as follows (Table S1): (i) three representatives from each 43 of 45 subfamilies currently established in CAZy (except for the subfamilies GH13_20 and GH13_45); (ii) ten representatives from the subfamily GH13_20 in order to cover all the specificities of this subfamily and to demonstrate clearly that the cyclomaltodextrinase from *Flavobacterium* sp. No. 92 and its counterparts do not belong to the neopullulanase subfamily GH13_20; and (iii) six representatives of the most recently established subfamily GH13_45—three members grouped around the α-amylase BaqA from *Bacillus aquimaris* [19,20] and the other three ones possessing the aberrant catalytic triad represented by the amylolytic enzyme BmaN1 from *Bacillus megaterium* [21]. Sequences from the individual GH13 subfamilies were selected with regard to as much information as possible available (i.e., experimental characterization and availability of three-dimensional structure were considered) in an effort to cover as many enzyme specificities as possible. The final studied set thus consisted of 253 sequences (Table S1) obeying the above-mentioned criteria.

All studied sequences were retrieved from the UniProt ([39]; https://www.uniprot.org/, accessed on 26 November 2022) and/or GenBank ([40]; https://www.ncbi.nlm.nih.gov/genbank/, accessed on 26 November 2022) databases.

### 2.2. Sequence Comparison and Evolutionary Analysis

Four different sequence alignments were performed using the program Clustal-Omega ([41]; https://www.ebi.ac.uk/Tools/msa/clustalo/, accessed on 26 November 2022). The first three alignments were executed on 108 *Flavobacterium* cyclomaltodextrinase-like sequences (that should define the new GH13 subfamily), whereas the fourth alignment was done with the full set of 253 sequences (i.e., including the additional 145 sequences covering all established GH13 subfamilies). While the former alignments (108 sequences) were based on: (i) N-terminal modules; (ii) the GH13 canonical part of the enzymes (i.e., catalytic TIM-barrel domain A including domains B and C); and (iii) the full-length enzymes; the latter alignment (253 sequences) was based on the substantial part of the catalytic domain spanning the sequence segment from beginning of the CSR-VI (strand β2) to the end of the CSRV-VII (strand β8) including the domain B. Information about the domain boundaries and all CSRs of individual sequences was obtained from the literature describing the sequences of biochemically characterized members of the individual subfamilies and previous in silico studies [10,17,19,20,21,22,23,24,30,31,32,33,34,42,43,44,45,46]. It is worth mentioning that in the case of the alignment of the full set of 253 sequences, in order to maximize sequence similarities, some manual tuning of the computer-produced alignment, especially with regard to CSRs, was necessary to perform.

Four evolutionary trees were calculated for the four above-mentioned sequence alignments. All were calculated as maximum-likelihood trees (including the gaps in the aligned sequences) using the WAG substitution model [47] and the bootstrapping procedure with 500 bootstrap trials [48], implemented in the MEGA software ([49]; https://www.megasoftware.net/, accessed on 29 November 2022). For the trees of the newly proposed GH13 subfamily, the branch swap filter parameter has been set to “very strong,” and all other specifications were used in a default mode. Finally, all four calculated tree files were displayed with the program iTOL ([50]; https://itol.embl.de/, accessed on 29 November 2022).

The sequence logo of seven well-established CSRs for all 108 sequences of the potentially novel GH13 subfamily represented by the cyclomaltodextrinase from *Flavobacterium* sp. No. 92 was created using the WebLogo 3.0 online server ([51]; http://weblogo.threeplusone.com/, accessed on 28 November 2022).

### 2.3. Comparison of Tertiary Structures and Docking Trials

Three-dimensional structures were retrieved from the Protein Data Bank (PDB; [52]; https://www.rcsb.org/, accessed on 11 November 2021) for (i) cyclomaltodextrinase from *Flavobacterium* sp. No. 92 ([24]; PDB code: 1H3G); (ii) selected representatives of all 45 GH13 subfamilies (Table S1); (iii) selected representatives of all fifteen CBM families considered as SBDs [14]; and (iv) GH13_5 α-amylase (AmyB) from *Halothermothrix orenii* ([43]; PDB code: 3BC9). For structural comparison of the *Flavobacterium* sp. No. 92 cyclomaltodextrinase N-terminal module with selected representatives of the fifteen SBD CBM families, the data were prepared to contain only the co-ordinates of the N-terminal cyclomaltodextrinase’s domain or an enzyme’s SBD from the particular CBM family by deleting the remaining parts of their structure based on the available literature [24,29,34,42,53,54,55,56,57]. In cases when no three-dimensional structure was available for any representative of a particular GH13 subfamily or for an SBD CBM family, structural models were created using the fold recognition server Phyre2 ([58]; http://www.sbg.bio.ic.ac.uk/~phyre2/, accessed on 11 November 2021).

The full-length tertiary structure of the cyclomaltodextrinase from *Flavobacterium* sp. No. 92 [24] was used for structural comparison with experimentally determined (if available) and modeled structures (if the real structure has not been solved as yet) of selected representatives of all 45 CAZy-defined GH13 subfamilies. Analogically, the tertiary structure of just the N-terminal domain of the cyclomaltodextrinase from *Flavobacterium* sp. No. 92 [24] was compared with experimental (if possible) or modeled structures of selected representatives of all 15 CBM families (CBM20, 21, 25, 26, 34, 41, 45, 48, 53, 58, 68, 69, 74, 82 and 83) recognized as SBDs [14] as well as with the N-terminal domain of the α-amylase AmyB from *Halothermothrix orenii* [43]. All tertiary structure comparisons were performed using the MultiProt server ([59]; http://bioinfo3d.cs.tau.ac.il/MultiProt/, accessed on 13 November 2021).

In order to inspect whether or not the N-terminal domain of the cyclomaltodextrinase from *Flavobacterium* sp. No. 92 may eventually function as an SBD. The docking trials using the program AutoDock and MGL Tools v1.5.6 [60] were performed. The dimeric structure of cyclomaltodextrinase ([24]; PDB code: 1H3G) was docked with α-, β- and γ-cyclodextrins. The protein structure and the substrates were prepared by adding polar hydrogen atoms and charges. The root of the ligand was detected using the torsion tree option. The grid map dimensions were set around the N-terminal domain, and all other parameters were set to default, and rigid docking was performed. Individual complexes were analyzed based on the Vina score, which represents the binding energy in kJ/mol. Three-dimensional structures of ligands (the α-, β- and γ-cyclodextrins) were retrieved from the PubChem database ([61]; https://pubchem.ncbi.nlm.nih.gov/, accessed on 30 September 2021) and converted into PDB coordinates by the SMILES program ([62]; https://cactus.nci.nih.gov/translate/, accessed on 30 September 2021). The resulting complexes of individual structures with bound cyclodextrins were displayed using the UCSF Chimera program [63].

## 3. Results

### 3.1. In Silico Analysis of the New Subfamily GH13_46

**Figure 1:**
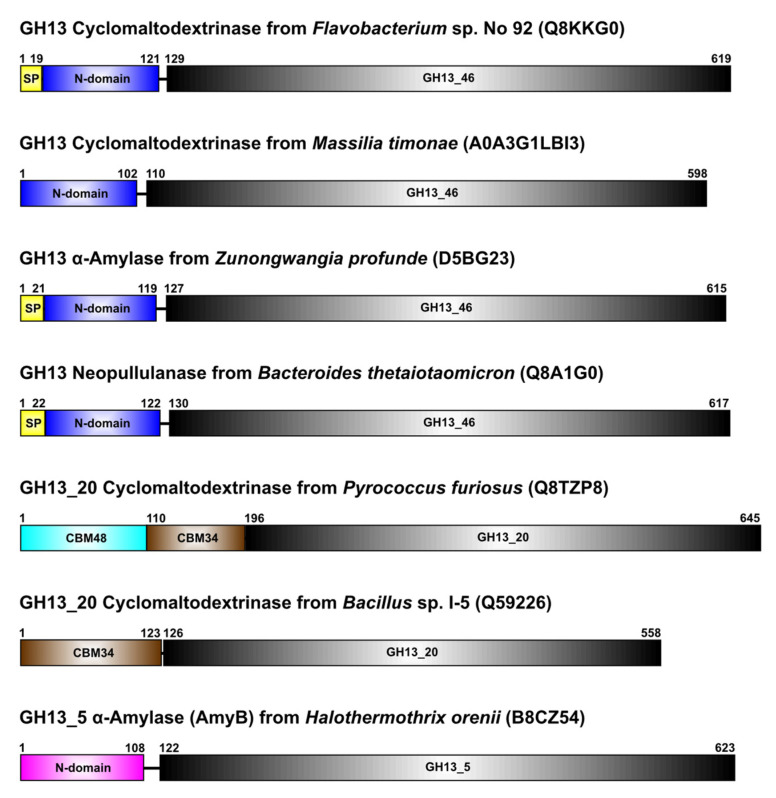
Domain arrangement of selected amylolytic enzymes of the present study. The four enzymes at the top are, until now, the only four biochemically characterized enzymes of the newly proposed GH13 subfamily GH13_46 represented by the cyclomaltodextrinase from *Flavobacterium* sp. No. 92. All of them contain an N-terminal domain preceding the catalytic GH13 TIM-barrel; currently, however, they are not assigned to any CBM family. In addition to them, two well-established cyclomaltodextrinases from the GH13_20 subfamily are shown to possess either the CBM34 (bacterial origin) or CBM48 and CBM34 (archaeal origin) at their N-termini. For comparison, the GH13_5 α-amylase AmyB from *Halothermothrix orenii,* also having a more unspecified N-terminal domain similar to those from the new subfamily, is presented.

Of 108 sequences proposed to constitute the novel GH13 subfamily (Table S1), only four have already been characterized as amylolytic enzymes: (i) cyclomaltodextrinase from *Flavobacterium* sp. No. 92 [24]; (ii) neopullulanase SusA from *Bacteroides thetaiotaomicron* [35]; (iii) α-amylase AmyZ from *Zunongwangia profunda* [36]; and (iv) cyclomaltodextrinase from *Massilia timonae* [37]. Their typical domain arrangement is illustrated in Figure 1.

In addition to family GH13 canonical three-domain composition, they possess a corresponding domain N at their N-terminus, just succeeding the signal peptide and preceding the catalytic TIM-barrel. Although this domain arrangement is similar to that characteristic of the functionally related subfamily GH13_20, the N-termini of those cyclomaltodextrinases are formed either, if of bacterial origin, by a single SBD of the family CBM34 or, if originated from archaeons, by two SBDs of families CBM48 and CBM34 in that order (Figure 1).

The amino acid sequence alignment of complete sequences of all 108 members of the newly proposed GH13 subfamily (Figure S1) clearly demonstrated their overall high similarity even if the N-terminal domain has been taken into account; the individual pair-wise sequence identities are shown in Table S2. Thus, the consensus length of the alignment counted 693 positions, the shortest and longest sequences being ranged from 588 to 616 residues (Figure S1), yielding the degree of sequence identity and similarity of 6.64% and 16.04%, respectively.

**Figure 2:**
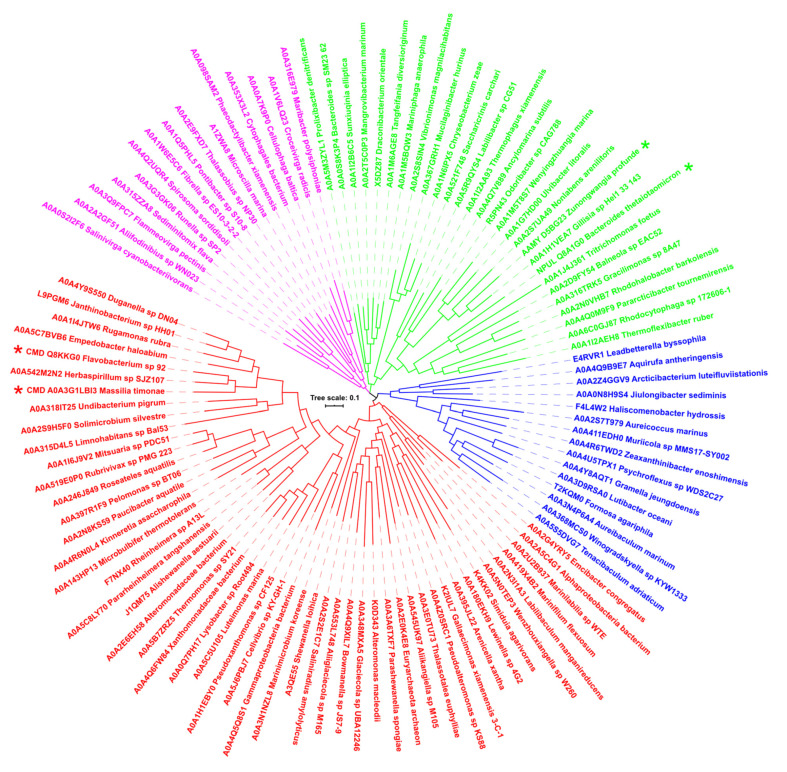
Evolutionary tree of the newly proposed GH13 subfamily GH13_46. The tree is based on the alignment (presented in Figure S1) of all 108 sequences of catalytic TIM-barrel (including the domain B) together with the N-terminal domain and domain C preceding and succeeding, respectively, the catalytic TIM-barrel. The labels of protein sources consist of the UniProt accession number and the name of the organism, the four experimentally characterized enzymes being marked by an asterisk. The four individual groups distinguished from each other by different colors correspond to representatives shown in Figure S1; the sequence order in the tree in an anticlockwise manner (starting from the first sequence in the red cluster) reflects their order in the alignment (starting from the top).

In order to follow the evolutionary relationships within the new GH13 subfamily, the evolutionary tree based on the alignment of complete sequences was calculated (Figure 2). Since of the 108 sequences compared, only four represent the experimentally characterized enzymes, i.e., two cyclomaltodextrinases, a neopullulanase, and an α-amylase, it is not easy to draw any relevant clues concerning the observed mutual relatedness among the sequences. Nevertheless, four clusters—two larger (50 and 28 sequences) and two smaller (15 sequences each)—can be seen in the tree, the four enzymes mentioned above being positioned in the two larger ones: the two cyclomaltodextrinases positioned in the 50-membered cluster, whereas both neopullulanase and α-amylase in the second cluster counting 28 members (Figure 2). Interestingly, the only eukaryotic representative from *Tritrichomonas foetus* (UniProt: A0A1J4J361), which is a microscopic single-celled flagellated protozoan parasite [64], has been located on a branch adjacent to the neopullulanase from *Bacteroides thetaiotaomicron* [35]. With regard to the only archaeal representative from *Euryarchaeota archaeon* (UniProt: A0A2E0K4E8), it has been placed in the largest cluster where the *Flavobacterium* sp. No. 92 cyclomaltodextrinase [24] is found, too, although at a rather long distance from it (Figure 2). With regard to the eventual enzyme activity/specificity of members of the newly proposed subfamily, it could only be deduced as a cyclomaltodextrinase (or neopullulanase) since the vast majority of the subfamily members (104 of 108) are represented by hypothetical proteins.

For comparison, two further corresponding evolutionary trees were constructed: (i) based on the alignment of family GH13 canonical domains A, B, and C, i.e., excluding the domain N (Figure S2); and (ii) based on the alignment of domain N, i.e., eliminating domains A, B, and C (Figure S3). It is worth mentioning that while in the former tree, the clustering of all 108 sequences copies to the substantial extent that is seen in the “whole-sequences” tree (Figure 2), in the latter tree, the sequences from the four original clusters are much more scattered indicating a different evolutionary rate for the N-terminal domain with respect to the remaining catalytic part of the sequence.

**Figure 3:**
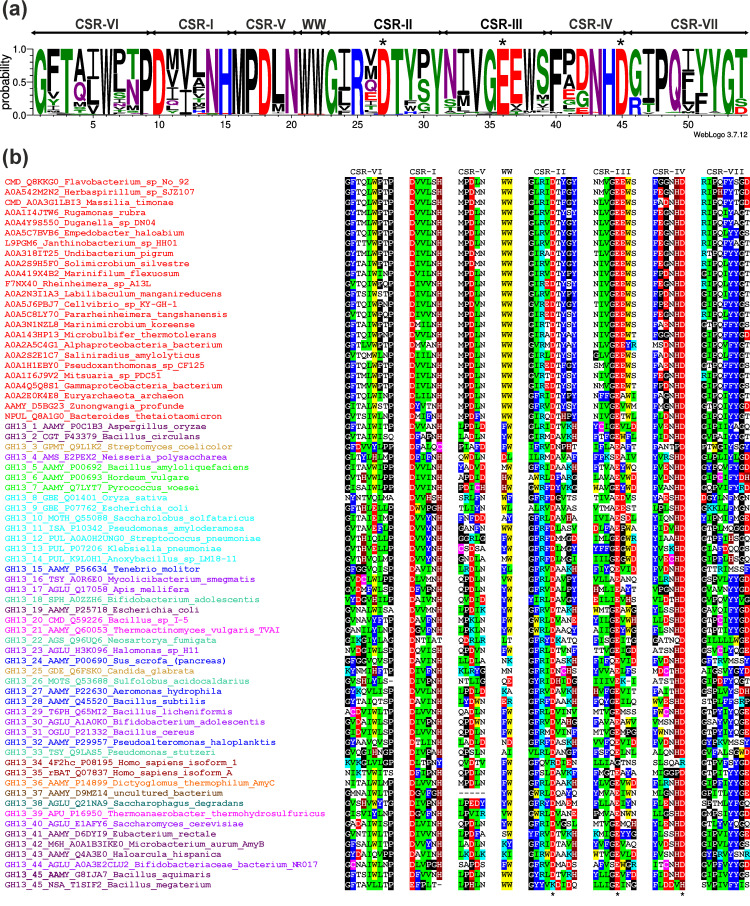
(**a**) Sequence logo of the newly proposed GH13 subfamily GH13_46. CSR-I, residues 10–15; CSR-II, residues 23–31; CSR-III, residues 32–39; CSR-IV, residues 40–45; CSR-V, residues 16–20; CSR-VI, residues 1–9; CSR-VII, residues 46–54; WW region, residues 21–22. The catalytic triad, i.e., the catalytic nucleophile (No. 27, aspartic acid), the proton donor (No. 36, glutamic acid), and the transition-state stabilizer (No. 45, aspartic acid) are indicated by asterisks. The logo is based on 108 sequences. (**b**) Conserved sequence regions for the α-amylase representatives. Twenty-four selected members (of all 108 studied in the logo) of the newly proposed GH13 subfamily represented by the cyclomaltodextrinase from *Flavobacterium* sp. No. 92 (red sources) and one sequence represented each GH13 subfamily (for details, please, see Table S1). Each protein is labeled by its UniProt accession number and the name of the organism. If known, the enzyme specificity is given preceded by the GH13 subfamily number. The abbreviations of the enzymes are explained in Table S1. The color code for the selected residues: W, yellow; F, Y—blue; V, L, I—green; D, E—red; R, K—cyan; H—brown; C—magenta; G, P—black. The catalytic triad is signified by asterisks below the CSRs.

In order to focus on the best-conserved segments of amino acid sequences of the newly proposed subfamily, all seven CSRs typical for the α-amylase family GH13 [2,10], including also the pair of consecutive tryptophans positioned in the helix α3 of the catalytic TIM-barrel [20] have been extracted from the alignment and presented as a sequence logo (Figure 3a). It is of note that despite the large size of the sample (108 sequences), many positions in the logo exhibit a high degree of conservation, if not even the invariance. Of the seven particular CSRs, the CSR-V may deserve special attention since it is conserved almost completely invariantly as MPDLN (positions 16–20 in the logo). In addition, the short stretch consisting of two tryptophans (between the CSR-V and CSR-II; position 21–22) is invariant in 107 of all 108 sequences of this novel subfamily; the only exception being observed as FW in the eukaryotic member from *Tritrichomonas foetus* (UniProt: A0A1J4J361; cf. Figure S1).

### 3.2. New Subfamily GH13_46 in the Overall α-Amylase Family GH13 Context

In addition to the inside analysis of the newly proposed subfamily, it is of particular interest to elucidate its relatedness to other GH13 subfamilies that have already been well-established. Although the members of the new subfamily evidently share all the seven CSRs characteristic of the α-amylase family GH13 [10], a few unique features can be traced there even at first glance (Figure 3b), the glutamic acid following the catalytic proton donor in the CSR-III (position 37 in the logo; Figure 3a) being obviously the most evident one (cf. also Figure S1). Furthermore, the “aromatic” end of the CSR-II (positions 29–31), consisting of two well-conserved tyrosines with either glycine, serine, or proline in the middle, is also a feature specific to the new subfamily (Figure 3). The two adjacent tryptophans between the CSR-V and CSR-II may be another pronounced feature of the new subfamily, but this stretch is also present in subfamily GH13_45. Finally, as far as the typical MPDLN sequence of the CSR-V is concerned, it is a feature characteristic also of the subfamily GH13_36 (Figure 3b).

**Figure 4:**
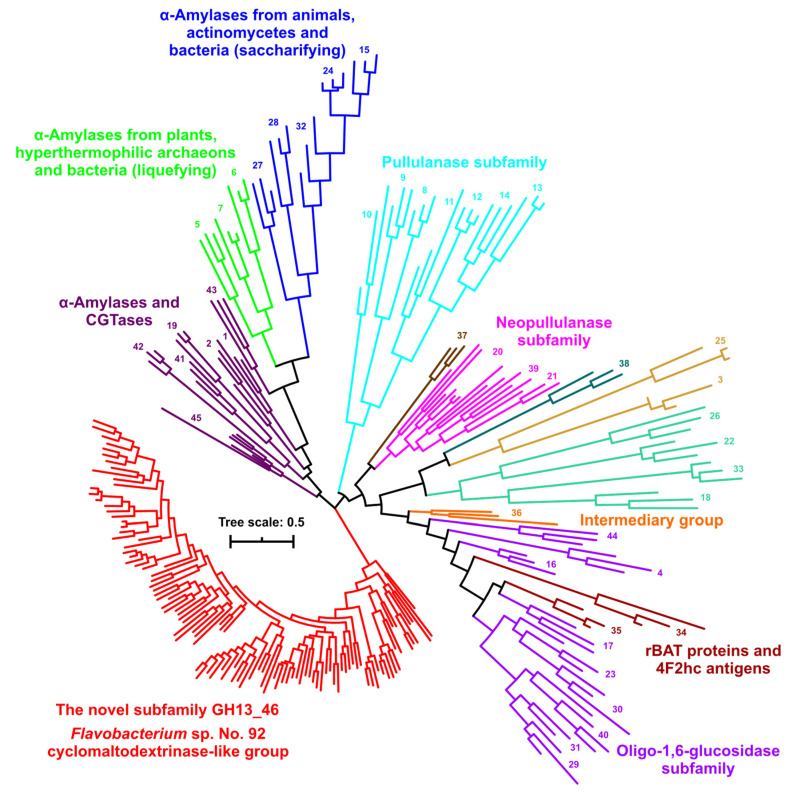
Evolutionary tree of the α-amylase family GH13. The tree covers 253 sequences with a focus on the novel subfamily around the cyclomaltodextrinase from *Flavobacterium* sp. No. 92 (for details, see Table S1). The tree is based on the alignment shown in Figure S4, spanning the sequence segment from the beginning of the strand β2 (CSR-VI) to the end of the strand β8 (CSR-VII), i.e., the substantial part of the catalytic TIM-barrel including the domain B. For the sake of simplicity, only the branches leading to the individual GH13 subfamilies, marked by their numbers, are shown. The same tree with all the leaves described is presented in Figure S5.

**Figure 5:**
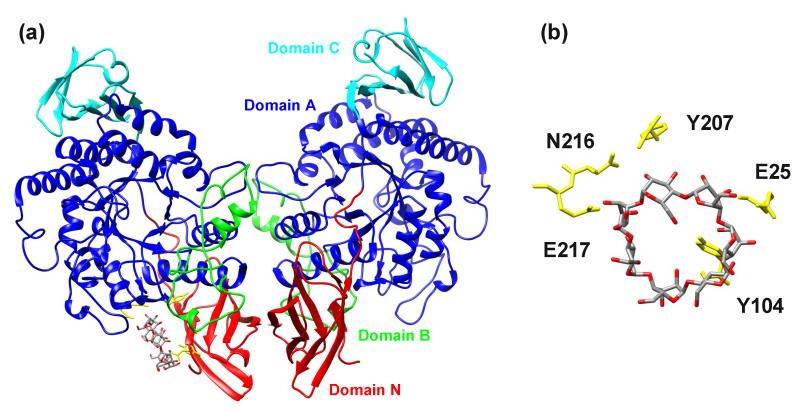
The visualization of the docking trials. (**a**) The dimeric structure of the cyclomaltodextrinase from *Flavobacterium* sp. No. 92 ([24]; PDB code: 1H3G) docked with β-cyclodextrin. (**b**) Detailed view of the potential binding site with a display of residues that could be involved in ligand binding. The individual domains are colored as follows: N-terminal domain—red; catalytic domain A—blue; domain B—green; domain C—cyan. For the sake of clarity, the numbering of the selected residues corresponds to the numbering of the amino acid sequence (UniProt: Q8KKG0), including the signal peptide. While the two residues, Glu25 and Tyr104, come from the N-terminal domain, the remaining three residues, Tyr207, Asn216, and Glu217, belong to the catalytic domain.

In spite of the fact that some conserved sequence features are shared with other GH13 subfamilies, the entire group of 108 sequences represented by the cyclomaltodextrinase from *Flavobacterium* sp. No. 92 evidently defines a novel subfamily of the α-amylase family GH13. This is most clearly demonstrated by the whole-family GH13 evolutionary tree (Figure 4) calculated based on the alignment of all 253 sequences of the present study (Table S1) spanning the substantial segment of the catalytic TIM-barrel, including domain B (Figure S4). Each already established GH13 subfamily (GH13_1-GH13_45) forms its own separate cluster in the tree; many particular subfamilies are grouped together into larger clusters due to their higher sequence similarities and closer evolutionary relatedness. It should be pointed out here that the tree shown in Figure 4 is a simplified tree with all the leaves removed and emphasizing just the existence of the novel GH13 subfamily. To see the details concerning all the sequences, the same tree—based on the same alignment (Figure S4)—has also been prepared as Figure S5. In any case, the newly proposed subfamily around the cyclomaltodextrinase from *Flavobacterium* sp. No. 92 is unambiguously separated from all the remaining GH13 subfamilies (Figure 5), which definitively justifies the assignment of this group a CAZy-curators-approved GH13 subfamily number—GH13_46.

### 3.3. Comparison of Tertiary Structures

In an effort to shed more light on the eventual position of the novel GH13 subfamily within the entire α-amylase family GH13, the experimentally determined three-dimensional structure of the cyclomaltodextrinase from *Flavobacterium* sp. No. 92 has been compared with representatives of each GH13 subfamily (Table S3). Since a tertiary structure is not available for each GH13 subfamily, structures of representative enzymes from those 12 subfamilies (without a real structure) have been obtained by homology modeling. In fact, structures of most established GH13 subfamilies—regardless of whether the experimentally determined structure or just a model—have resulted in a reasonable superposition with the structure of the cyclomaltodextrinase with 300–350 corresponding Cα atoms and the root-mean-square deviation around 1.50 Å (Table S3). Nevertheless, the best data from the individual overlays have been obtained for comparison of the *Flavobacterium* sp. No. 92 cyclomaltodextrinase with representatives of subfamilies GH13_20, GH13_21, and GH13_39 forming the so-called neopullulanase subfamily (Figure 4). This indicates that the members of those three subfamilies—also containing cyclomaltodextrinases and/or functionally related neopullulanases—may represent the closest structural relatives of the members of the newly proposed subfamily.

Because of the positional resemblance of the N-terminal domain of the cyclomaltodextrinase from *Flavobacterium* sp. No. 92 with the N-terminal SBD of the family CBM34 present in their counterparts from subfamilies GH13_20 (Figure 1), the subsequent structural comparison has been focused on the isolated domain N of the cyclomaltodextrinase. Of 15 well-established SBD CBM families [14], except for the CBM74, which is approximately 3 times longer and no tertiary structure is available for it, representatives of all remaining 14 SBD CBM families were used for comparison. Interestingly, the results for all pair-wise superimpositions—again regardless of whether for the experimentally solved structure or just for a model—have been found as more-or-less similar to each other (Table S4). In other words, for the N-terminal domain of the cyclomaltodextrinase, no substantially higher structural similarity has been observed to any known SBD CBM family (for most cases, 30–45 corresponding Cα atoms with the root-mean-square deviation between 1.80–2.00 Å). This is in agreement with the fact that the domain N of the cyclomaltodextrinase has not been classified into any SBD CBM family as yet. It, however, makes sense to point out that a remarkably better structural overlay has been observed with the N-terminal domain of the α-amylase AmyB from *Halothermothrix orenii* (62 corresponding Cα atoms and the root-mean-square deviation 1.77 Å; Table S4) that, until now, similarly has not been classified to any existing SBD CBM family [14].

### 3.4. Docking Trials

In order to verify whether the N-terminal domain of members of the newly suggested GH13 subfamily could possess a carbohydrate-binding function and thus eventually represent a new CBM family, docking trials were performed. The dimeric structure of the cyclomaltodextrinase from *Flavobacterium* sp. No. 92 ([24]; PDB code: 1H3G) was docked with α-, β- and γ-cyclodextrins. In all three cases, the blind docking with the grid box targeted on the N-terminal domain indicated the same potential binding site with the score favoring the binding in the order α-, γ- and β-cyclodextrins, i.e., −5.2, −5.7 and −6.4 kJ/mol, respectively. The potential binding site could be formed around the Tyr104 (*Flavobacterium* sp. No. 92 cyclomaltodextrinase numbering, including the signal peptide), which can provide possible stacking interactions (Figure 5). It should be taken into account. However, the position of Tyr104 is not conserved invariantly in the domain N; therefore, its eventual role in carbohydrate binding can hardly be generalized for the entire newly proposed GH13 subfamily. Nevertheless, in all three studied cases (α-, γ- and β-cyclodextrins), the residues of the catalytic domain have also been found involved in ligand binding by providing hydrogen bonds. This suggests that the potential binding site could be arranged by residues coming from both the N-terminal and the catalytic domains.

## 4. Discussion

The cyclomaltodextrinase from *Flavobacterium* sp. No. 92 has been identified and characterized in fundamental studies published already in 1993–1994 [25,26,27,28], and its three-dimensional structure has also been known for almost 20 years [24]. It has therefore been justified to consider why, over the decades, not enough attention has been devoted to its detailed sequence-structural analysis in order to either include it in one of the already established GH13 subfamilies or—if that is not possible—to create a new GH13 subfamily.

This enzyme may really be of special interest. It exhibits the enzyme specificity of a cyclomaltodextrinase (EC 3.2.1.54) that is typically from the CAZy subfamily GH13_20 [16] and is nearly indistinguishable from both maltogenic amylase and neopullulanase [31,32,33], grouped together in the so-called neopullulanase subfamily [22]. The cyclomaltodextrinase from *Flavobacterium* sp. No. 92 possesses, however, several sequence-structural features that have prevented adding this enzyme to ordinary GH13_20 members, especially: (i) the N-terminal domain not compatible with the SBD of the family CBM34 (Figure 1) present in GH13_20 cyclomaltodextrinases [30]; and (ii) the sequence MPDLN in the CSR-V highly typical for amylolytic enzymes from the subfamily GH13_36 (Figure 3, Figures S1 and S4), for which this stretch has been used as a specific sequence marker [22,23]. All these attributes can be assigned not only to three closely related experimentally characterized counterparts, i.e., the neopullulanase SusA from *Bacteroides* thetaiotaomicron [35], the α-amylase AmyZ from *Zunongwangia profunda* [36] and the cyclomaltodextrinase from *Massilia timonae* [37] but also to a relatively robust group of more than 100 hypothetical proteins almost completely of a sole bacterial origin (Table S1).

With regard to relationships within the new subfamily, three evolutionary trees have been constructed based on the alignment of (i) complete sequences (Figure 2); (ii) sequences of catalytic TIM-barrel, domain B and domain C (Figure S2); and (iii) sequences of domain N (Figure S3). While four potential groups could be traced in the “whole-sequences” tree (Figure 2), which have partially been identifiable also in the tree reflecting the TIM-barrel with domains B and C (Figure S2), the tree calculated from the alignment of the isolated domain N has displayed a different clustering for the majority of sequences (Figure S3). This is, however, not too surprising since a similar behavior has been demonstrated previously for non-catalytic domains of amylolytic enzymes, often for various SBD CBM families and even for those from the neopullulanase subfamily [14,30,65,66,67].

The proposal to establish a novel GH13 subfamily around the cyclomaltodextrinase from *Flavobacterium* sp. No. 92 may strongly be supported by a comparison of all seven CSRs (Figure 3) characteristic of the α-amylase family GH13 [2,10], but it is best evident from the clustering of all 253 sequences studied here (Table S1) in the evolutionary tree (Figure 4). The tree—its detailed version is illustrated in Figure S5—has been based on the sequence alignment spanning the substantial part of the catalytic TIM-barrel, including the domain B (Figure S4). First, it has confirmed the mutual relatedness among the individual GH13 subfamilies described by numerous previous in silico studies, such as, e.g., oligo-1,6-glucosidase and neopullulanase subfamilies [22,23], rBAT proteins and 4F2hc antigens [68], pullulanase subfamily [69], α-amylases from plants and archaeons [42], α-amylases from animals and actinomycetes [70], α-amylases from different fungi [71], and others. However, what is more, important with regard to the present study, it has convincingly shown the branch leading to the cluster of the novel GH13 subfamily clearly separated from the remaining subfamilies (Figure 4). The compactness of the proposed new subfamily in terms of sequence similarity is also supported by the sequence logo (Figure 3a) that, in spite of a quite large number of sequences (108 proteins; cf. Table S1), contains many individual positions and short stretches as invariantly or at least highly conserved. Although some of them—e.g., the sequence MPDLN in the CSR-V (positions 16–20 in the logo) and the two adjacent tryptophans between CSR-V and CSR-II (positions 21–22)—have been found to be shared with other GH13 subfamilies, i.e., GH13_36 [22,23] and GH13_45 [19,20,21], respectively, some others—such as the “aromatic” end of the CSR-II (positions 29–31) and the well-conserved glutamic acid just following the catalytic proton donor in the CSR-III (position 37)—have been identified to be unique for this novel GH13 subfamily (Figure 3b).

As far as the structural comparison of the full-length cyclomaltodextrinase from *Flavobacterium* sp. No. 92 [24] with its counterparts representing the individual established GH13 subfamilies, the data have not revealed any especially pronounced close pair-wise homology (Table S3). Despite the separated position of the entire cluster of the novel subfamily in the evolutionary tree (Figure 4), from the structural point of view, it is possible to point out that the members of the so-called neopullulanase subfamily [22,30] could be considered the most closely related structural homologs of the new subfamily. They are represented here by various enzyme specificities from CAZy subfamilies GH13_20 and GH13_21 [31,32,33,34,72,73,74,75,76,77,78], and eventually GH13_39 (Table S3). However, since the *Flavobacterium* sp. No. 92 cyclomaltodextrinase shares no exceptionally high level of structural similarity with any GH13 subfamily. This fact also supports the independence of the entire group it represents as a novel subfamily.

In the following part of the structural analysis of the cyclomaltodextrinase, attention was aimed at its N-domain itself. The results from the pair-wise comparisons of this domain with representatives of all 14 relevant SBD CBM families [14] support the fact that the domain N of the cyclomaltodextrinase is currently not a member of any existing CBM family since no reasonably high structural similarity has been observed (Table S4). Moreover, there is no evidence that the N-domain may be involved in binding cyclodextrins (or α-glucans in general) by the cyclomaltodextrinase from *Flavobacterium* sp. No. 92, nor the tertiary structures solved to date have been determined with any α-glucan bound to the domain N [24,29]. It, therefore, still cannot define a novel CBM family in the CAZy database [1], although its overall structure adopts an immunoglobulin-like fold [24] typical for an SBD of amylolytic enzymes [14]. Interestingly, the domain N was shown to be involved in the oligomerization of the cyclomaltodextrinase, which typically exists as a loose dimer of tight dimers; the Thr49 being identified as the residue responsible for the loose contact of dimers [29]. Threonine is, however, not highly conserved throughout the newly proposed GH13 subfamily (Figure S1). It is worth mentioning that the spatial arrangement of individual monomers in dimer and/or even the tetramer [29] does not preclude the potential saccharide binding by the domain N, as observed in our docking trials (Figure 5). Regardless of the above-mentioned facts, the N-domain of the cyclomaltodextrinase shares a substantially higher structural similarity with the CBM-like N-terminal domain of the GH13_5 α-amylase AmyB from *Halothermothrix orenii* (Figure 1, Table S4). Again, neither this potential SBD, even being demonstrated to be responsible for the binding of AmyB to raw corn starch [43], has not been assigned any CBM family until now [1,14].

The presented in silico experiments were finally completed by docking trials performed in an effort to demonstrate the eventual binding of α-glucans by the N-terminal domain of the cyclomaltodextrinase from *Flavobacterium* sp. No. 92. A similar CBM-like domain of amylomaltases from *Escherichia coli* [79] and *Corynebacterium glutamicum* [80] from the family GH77 was recently analyzed and based on docking of maltooligosaccharides to their N-terminal domain, predicted to represent a new type of SBD and define a new CBM family [81]. Here, for all the three docked α-glucans, i.e., α-, β- and γ-cyclodextrins, a reasonable binding has been detected, the most favorable score −6.4 kJ/mol being observed for β-cyclodextrin (Figure 5). As the most prominent residue potentially involved in a single binding site, Tyr104 of the cyclomaltodextrinase has been identified. In general, a crucial binding residue of any SBD CBM family should be capable of providing stacking interactions [14]. Although the position of the Tyr104 is not conserved invariantly throughout the newly proposed subfamily (Figure S1), its aromatic character, if conserved, makes it, in principle, feasible. These predictions thus have to be verified experimentally, but the presented bioinformatics analysis could stimulate the acceleration of research focused on the N-terminal domain as a potential CBM.

In any case, based on the present study, the entire group of amylolytic enzymes and hypothetical proteins represented by the cyclomaltodextrinase from *Flavobacterium* sp. No. 92 definitively deserves the creation of its own new subfamily within the α-amylase family GH13, the subfamily GH13_46.

## Data Availability

Not applicable.

## Supplementary Materials

The following are available online at https://www.mdpi.com/article/10.3390/molecules27248735/s1: Figure S1: Sequence alignment of the newly proposed subfamily GH13_46 represented by the cyclomaltodextrinase from *Flavobacterium* sp. No. 92. Figure S2: Evolutionary tree of the newly proposed subfamily GH13_46. Figure S3: Evolutionary tree of the newly proposed subfamily GH13_46. Figure S4: Sequence alignment of the α-amylase family GH13. Figure S5: Evolutionary tree of the α-amylase family GH13. Table S1: List of all 253 sequences of the present study. Table S2: Matrix of the pair-wise identity (%) of 108 sequences of the newly proposed subfamily GH13_46. Table S3: Tertiary structure comparison of the full-length cyclomaltodextrinase from *Flavobacterium* sp. No. 92 with representatives of individual GH13 subfamilies. Table S4: Tertiary structure comparison of the N-terminal domain of cyclomaltodextrinase from *Flavobacterium* sp. No. 92 with individual representatives of real SBD CBM families.

## Abbreviations

CAZy, Carbohydrate-Active enZymes; CBM, carbohydrate-binding module; CSR, conserved sequence region; GH, glycoside hydrolase; PDB, Protein Data Bank; SBD, starch-binding domain.
