# Supplementary material for: A Novel Subfamily GH13_46 of the α-Amylase Family GH13 Represented by the Cyclomaltodextrinase from Flavobacterium sp. No. 92
Source: Molecules. 2022 Dec 9;27(24):8735. doi: 10.3390/molecules27248735 (PMC9781549; doi:10.3390/molecules27248735)
Supplement: Supplementary file 1 [file Supplementary_data_Janecek_Figure_S2_R1.pdf › Supplementary_data_Janecek_Figure_S2_R1.pdf]

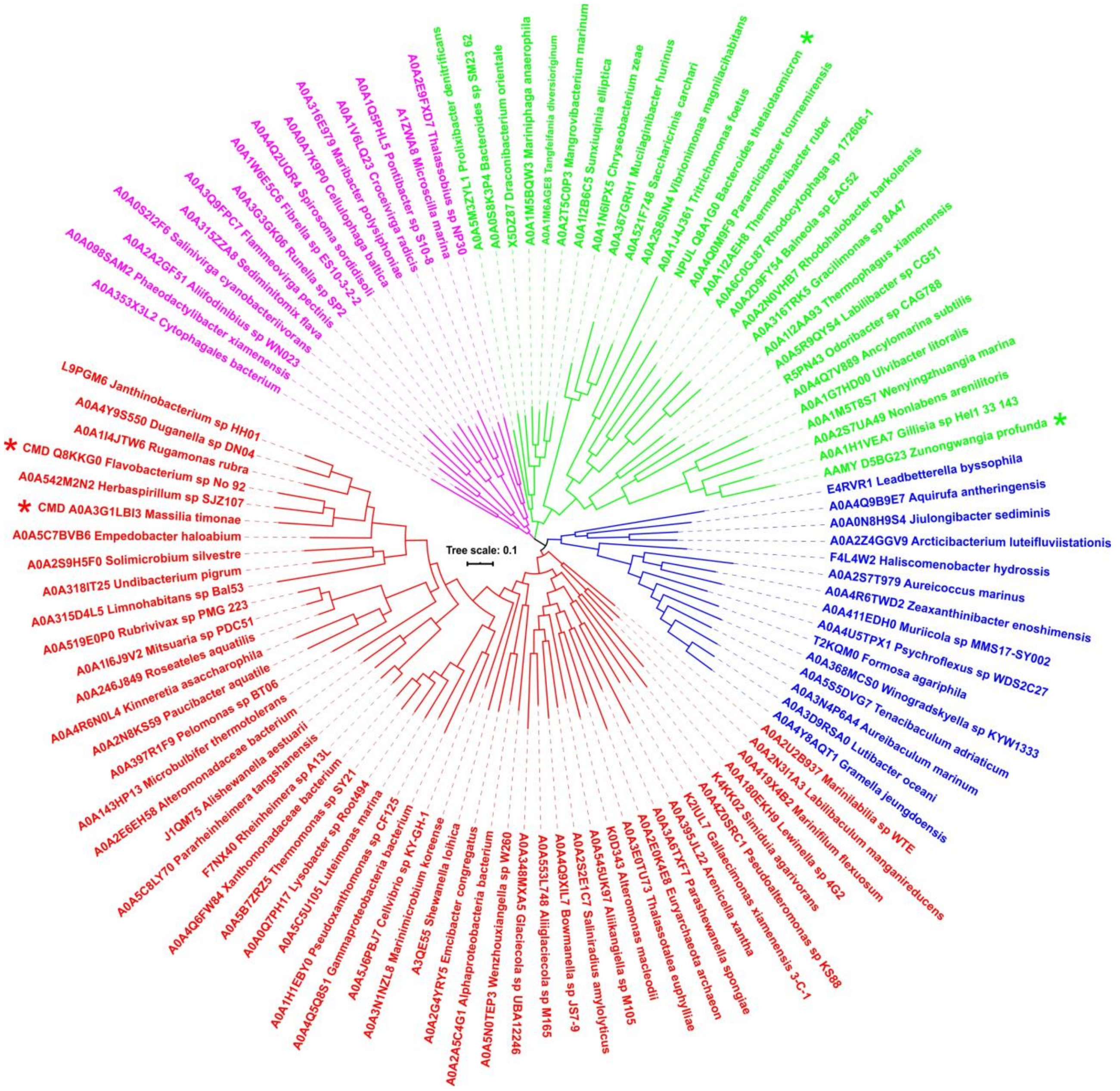

Figure S2. Evolutionary tree of the newly proposed subfamily GH13_46. The tree is based on the alignment of all 108 sequences of catalytic TIM-barrel (including the domain B) together with the succeeding domain C (the alignment can be inspected in Figure S1). The sequences are coloured according to four clusters seen in Figure 2 based on the alignment of entire sequences (Figure S1). The labels of protein sources consist of the UniProt accession number and the name of the organism (for details, see Table S1). The four experimentally characterized enzymes are marked by an asterisk.
