# Supplementary material for: A Novel Subfamily GH13_46 of the α-Amylase Family GH13 Represented by the Cyclomaltodextrinase from Flavobacterium sp. No. 92
Source: Molecules. 2022 Dec 9;27(24):8735. doi: 10.3390/molecules27248735 (PMC9781549; doi:10.3390/molecules27248735)
Supplement: Supplementary file 1 [file molecules-27-08735-s001.zip › Supplementary_data_Janecek_Figure_S1_R1.pdf]

Figure S1. Sequence alignment of the newly proposed subfamily GH13_46 represented by the cyclomaltodextrinase from *Flavobacterium* sp. No. 92. The alignment of all 108 selected members (Table S1) covers their complete sequences, i.e. the canonical family GH13 domains A (catalytic TIM-barrel), B (inserted roughly between CSR-I and CSR-II) and C (succeeding the CSR-VII) as well as the domain N (preceding the catalytic TIM-barrel). All seven family GH13 CSRs are boxed by black frames, the N-terminal domain is indicated by the red horizontal lane above the alignment. The residues of the catalytic triad are located in CSR-II (aspartic acid), CSR-III (glutamic acid) and CSR-IV (aspartic acid). Identical and similar positions are signified by asterisks and dots/semicolons under the alignment blocks. The colour code for the selected residues: W, yellow; F, Y – blue; V, L, I – green; D, E – red; R, K – cyan; H – brown; C – magenta; G, P – black. The labels of protein sources consist of the UniProt accession number and the name of the organism, the four experimentally characterized enzymes being marked by an asterisk. The four individual groups distinguished from each other by different colours correspond to representatives shown in Figure 2; the sequence order in the alignment (starting from the top) reflects their order in the tree in the anticlockwise manner (starting from the first sequence in the red cluster).

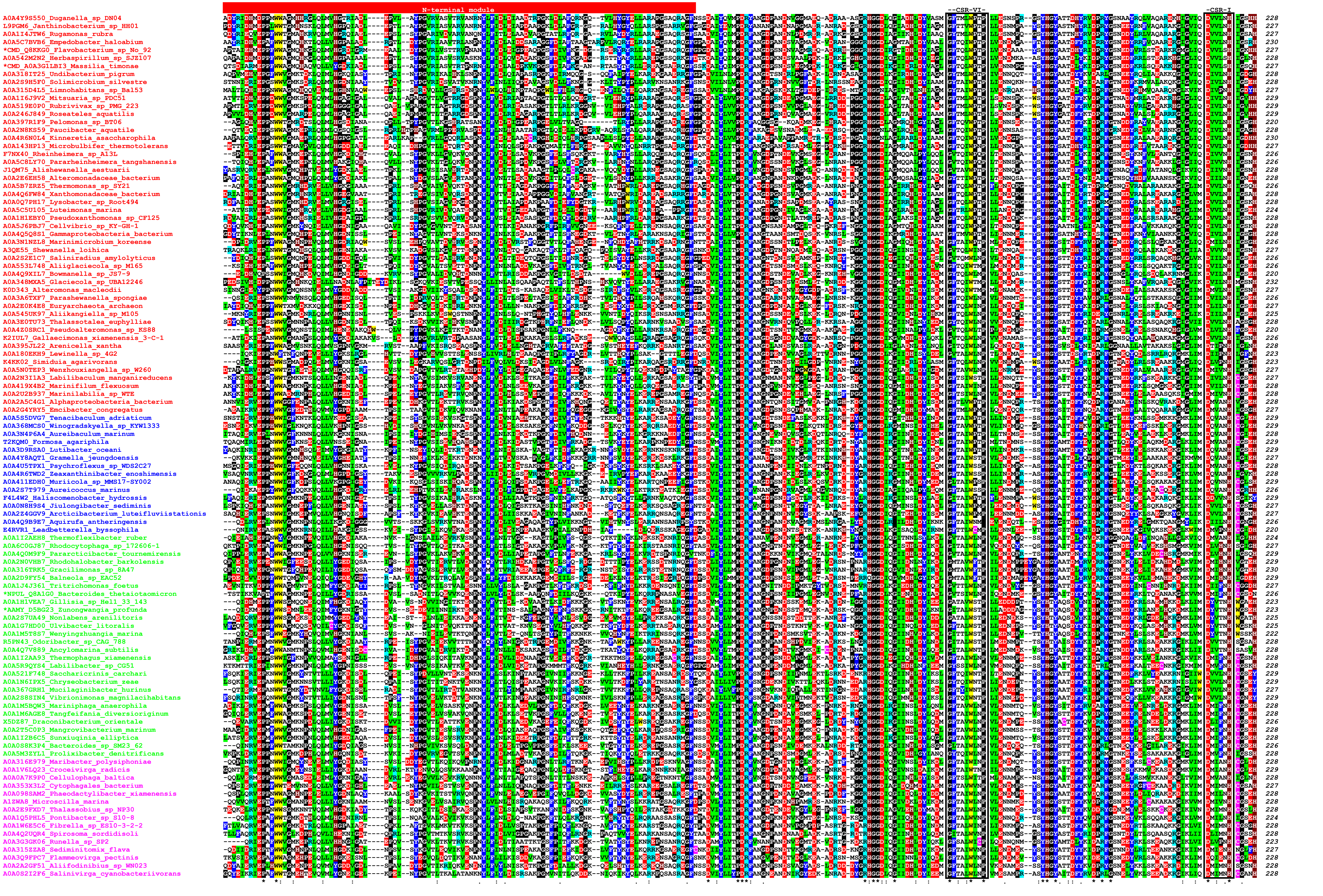
N-terminal module
--CSR-VI-
-CSR-I

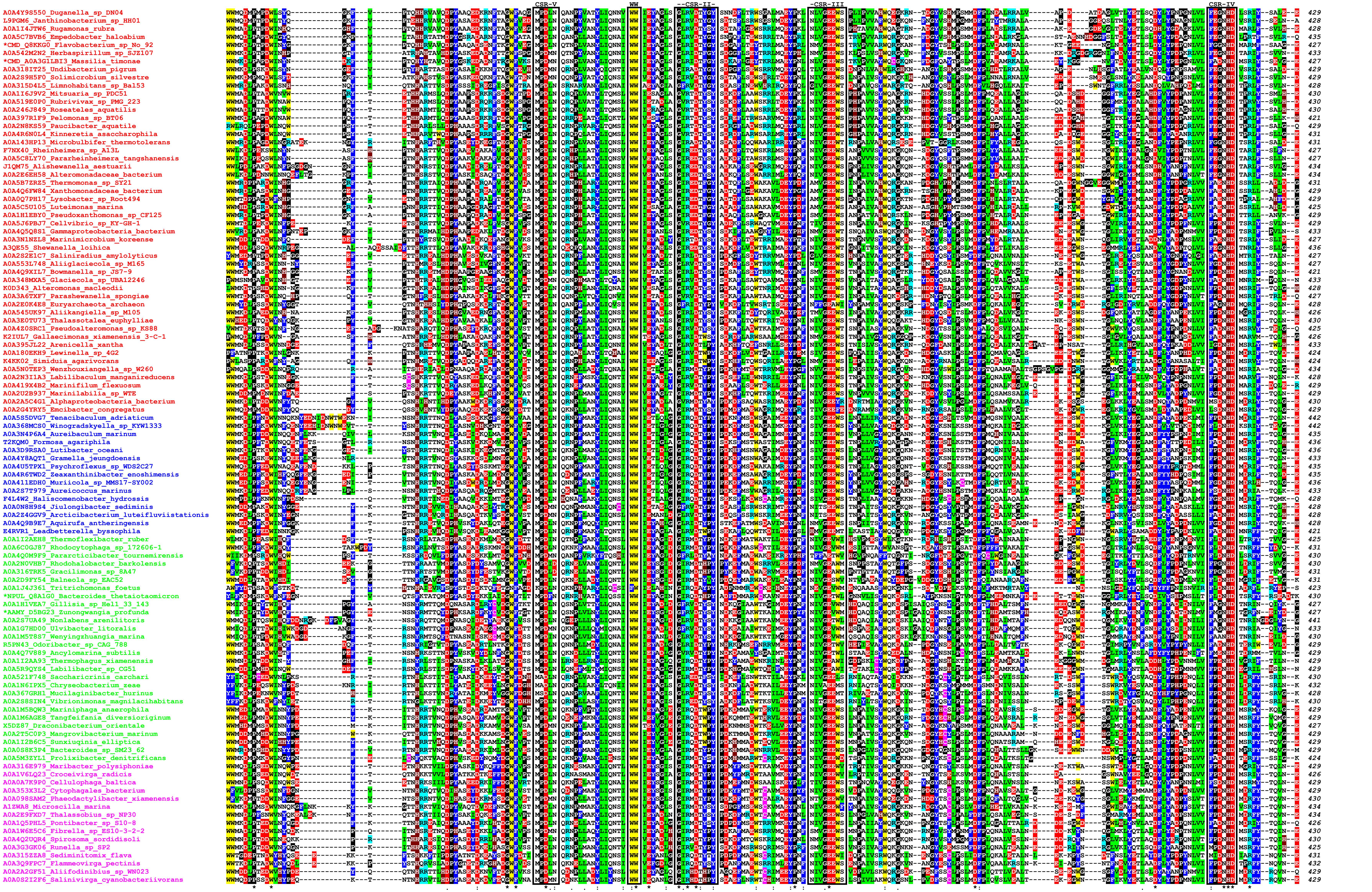
CSR-V
WW
--CSR-II-
-CSR-III
CSR-IV

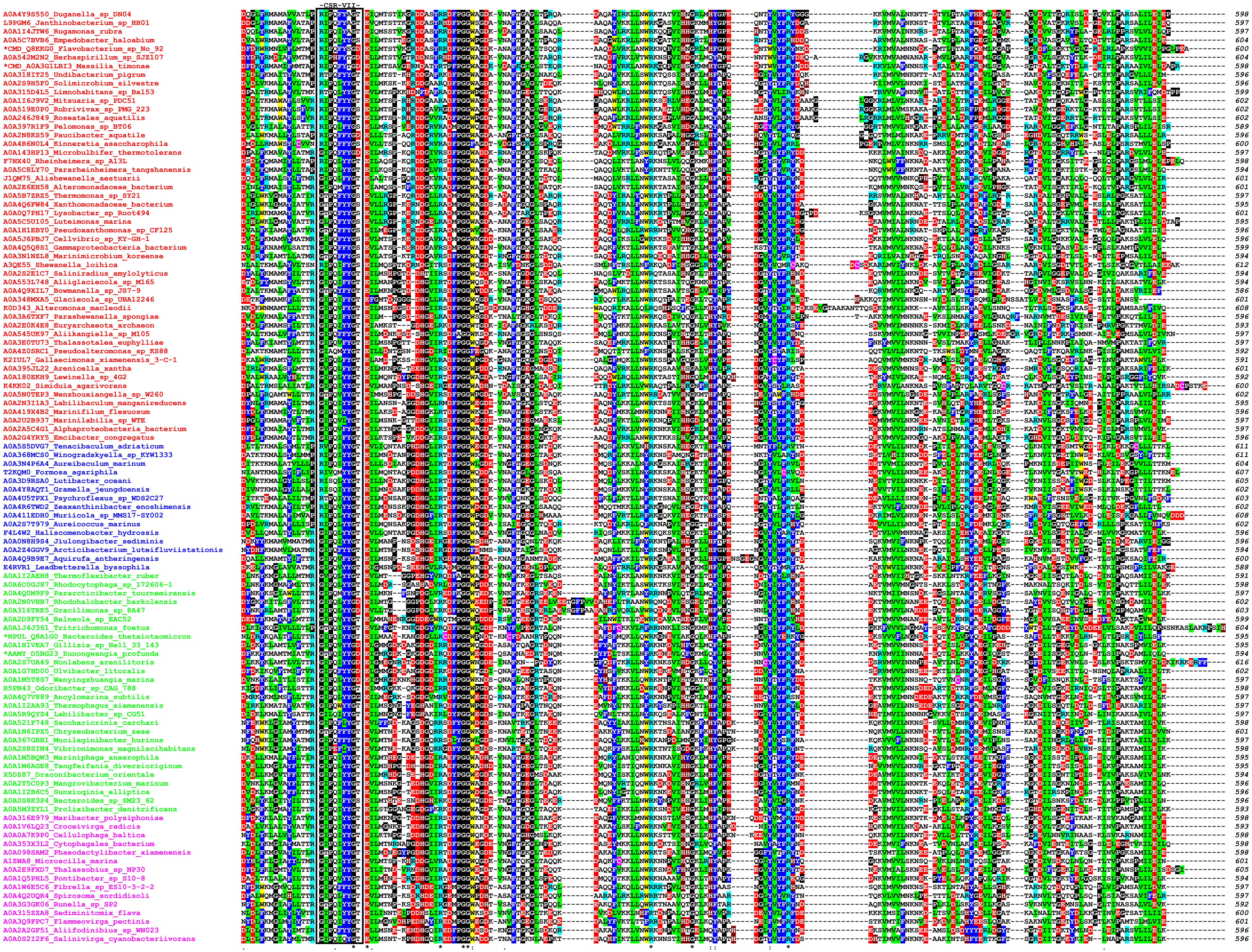
-CSR-VII-
