# Supplementary material for: A Novel Subfamily GH13_46 of the α-Amylase Family GH13 Represented by the Cyclomaltodextrinase from Flavobacterium sp. No. 92
Source: Molecules. 2022 Dec 9;27(24):8735. doi: 10.3390/molecules27248735 (PMC9781549; doi:10.3390/molecules27248735)
Supplement: Supplementary file 1 [file molecules-27-08735-s001.zip › Supplementary_data_Janecek_Figure_S4_R1.pdf]

Figure S4. Sequence alignment of the α-amylase family GH13. The alignment delivers 253 sequences with focus on the novel subfamily GH13_46 around the cyclomaltodextrinase from *Flavobacterium* sp. No. 92 (for details, see Table S1). The alignment spans the sequence segment from the beginning of the strand β2 (CSR-VI) to the end of the strand β8 (CSR-VII), i.e. substantial part of the catalytic TIM-barrel including the domain B. All seven family GH13 CSRs are boxed by black frames. The colour code for the selected residues: W, yellow; F, Y – blue; V, L, I – green; D, E – red; R, K – cyan; H – brown; C – magenta; G, P – black. The numbers at the end of the alignment correspond with the length of aligned sequence segments.

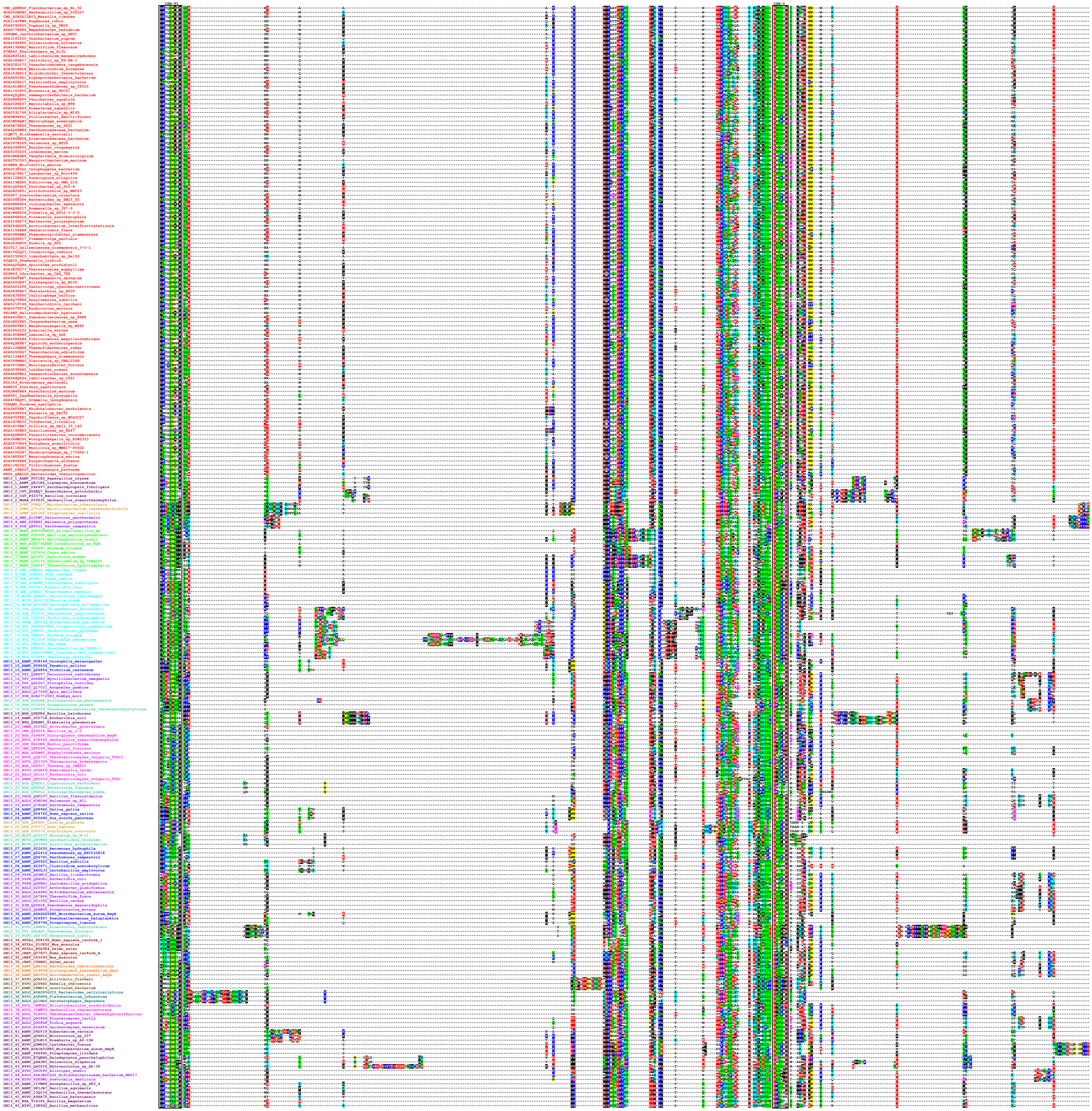
CSR-VI
CSR-I

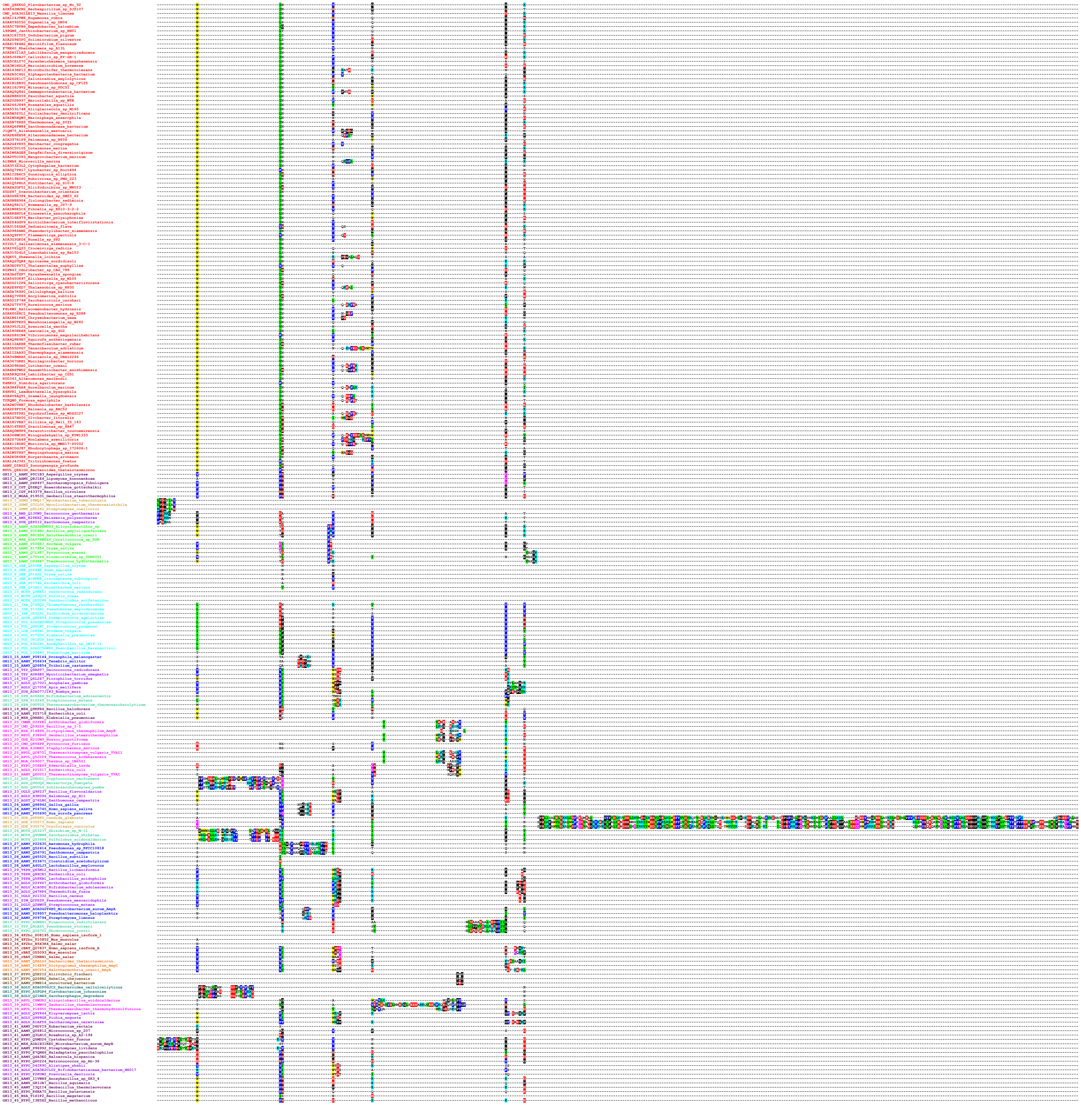

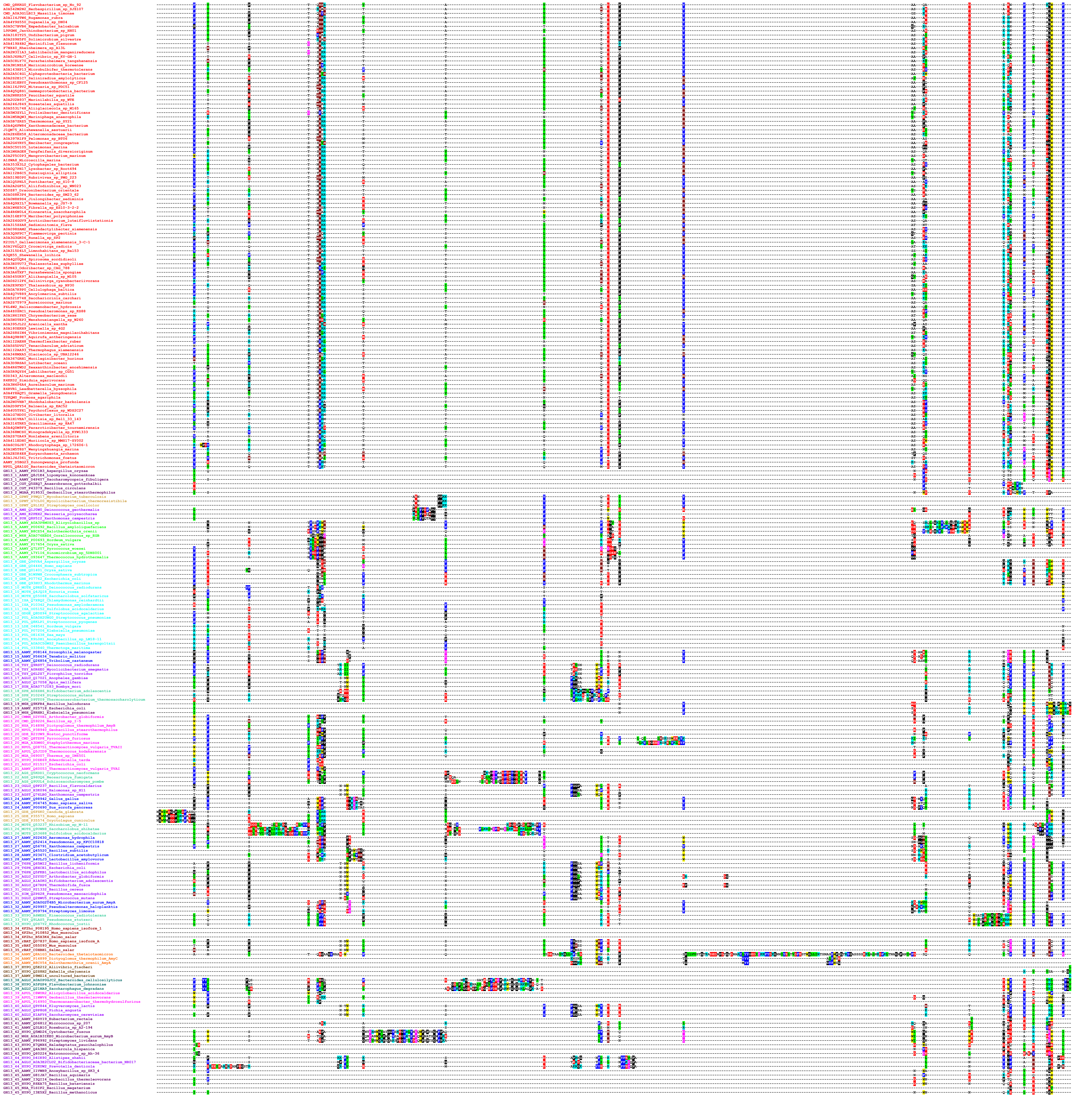

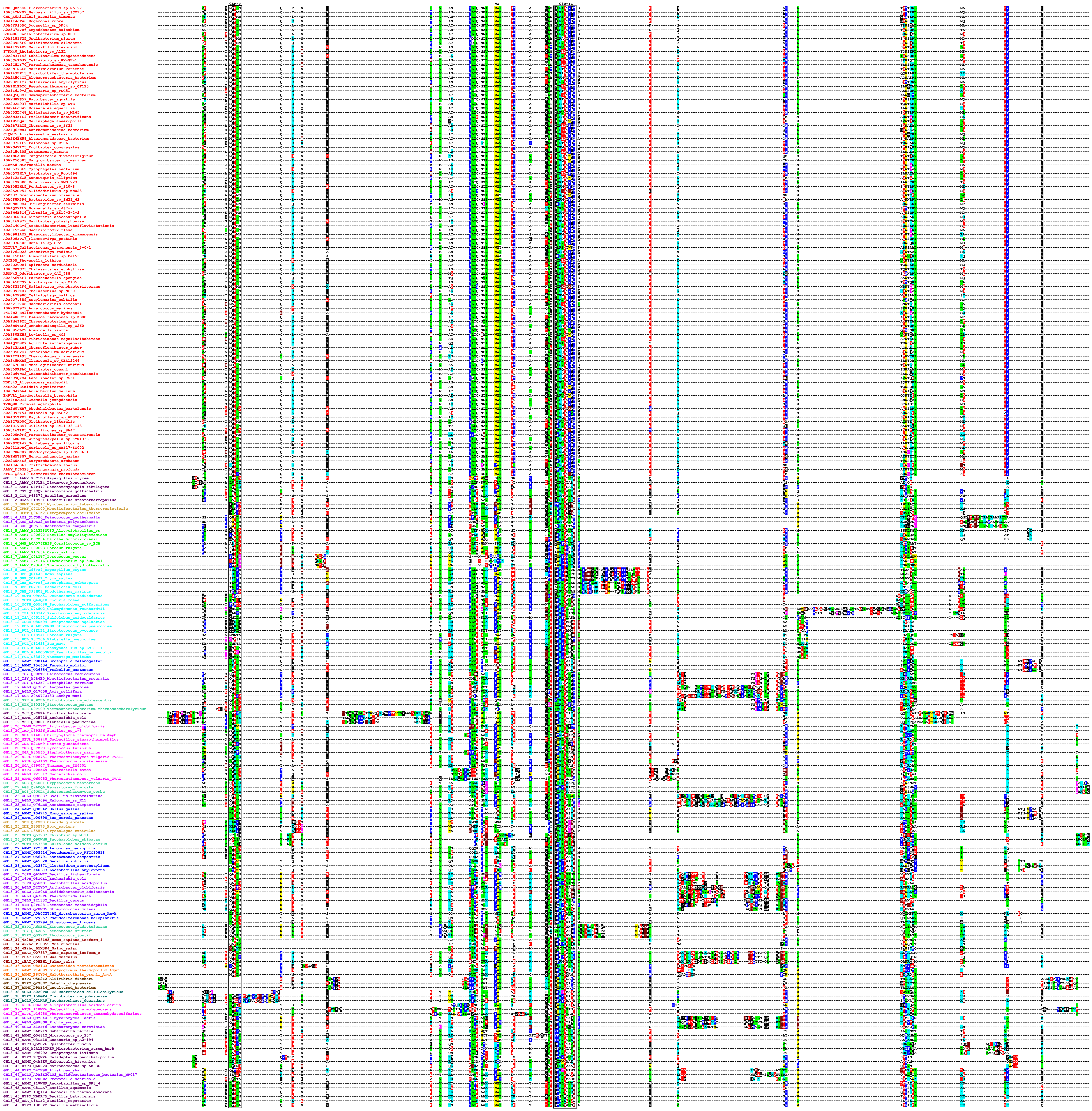
CSR-V
WW
CSR-II

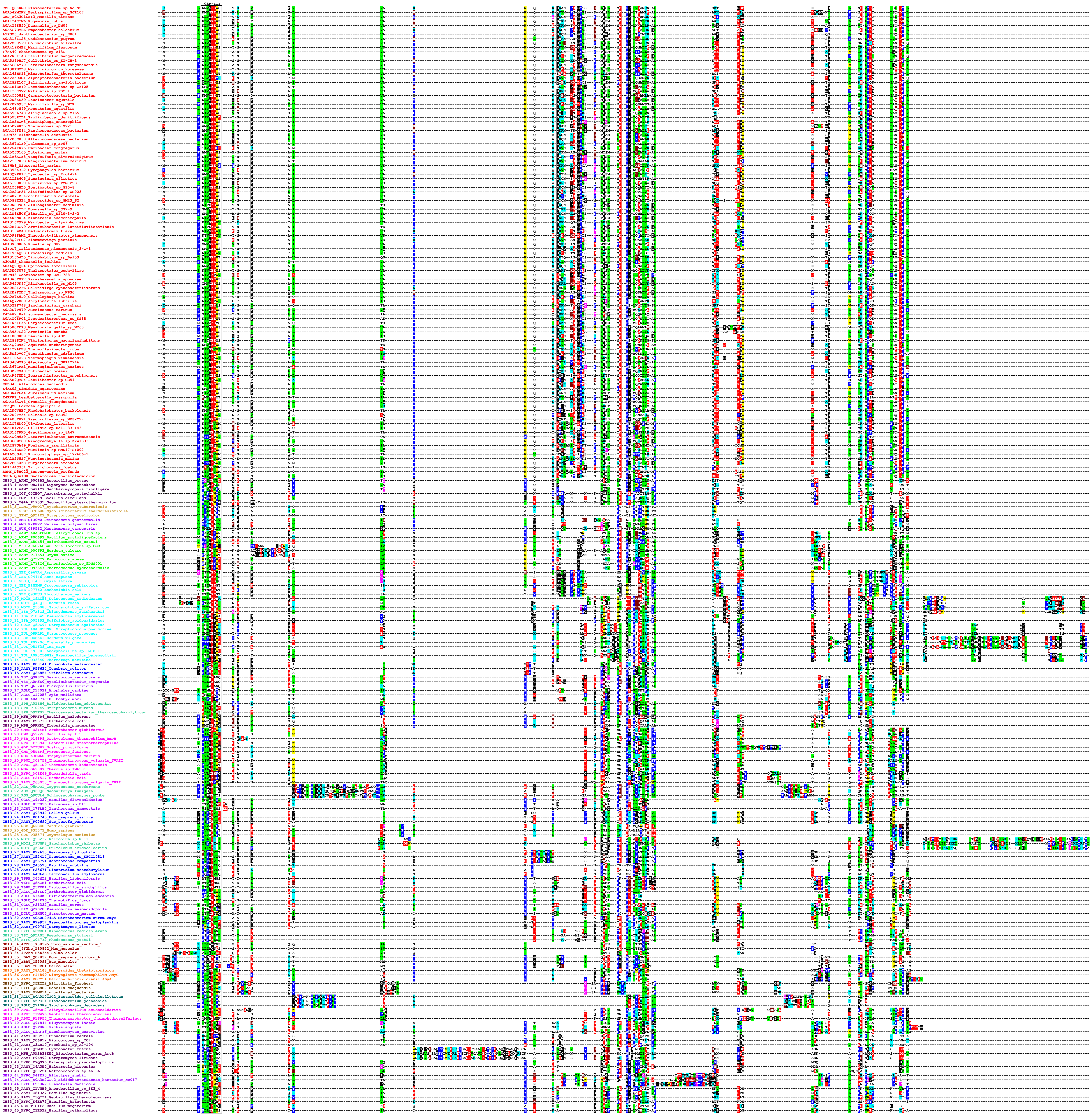

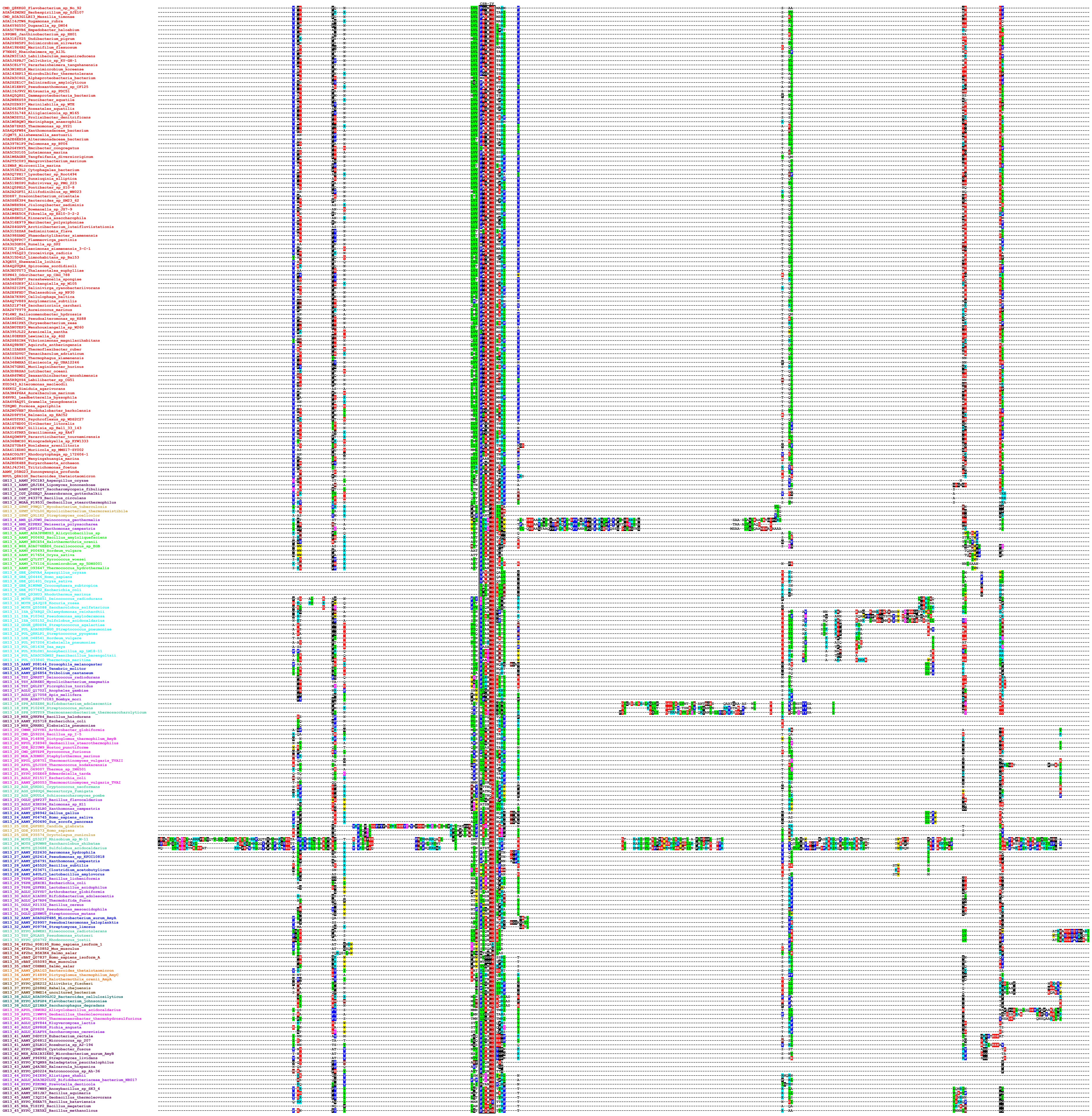
CBS-IV

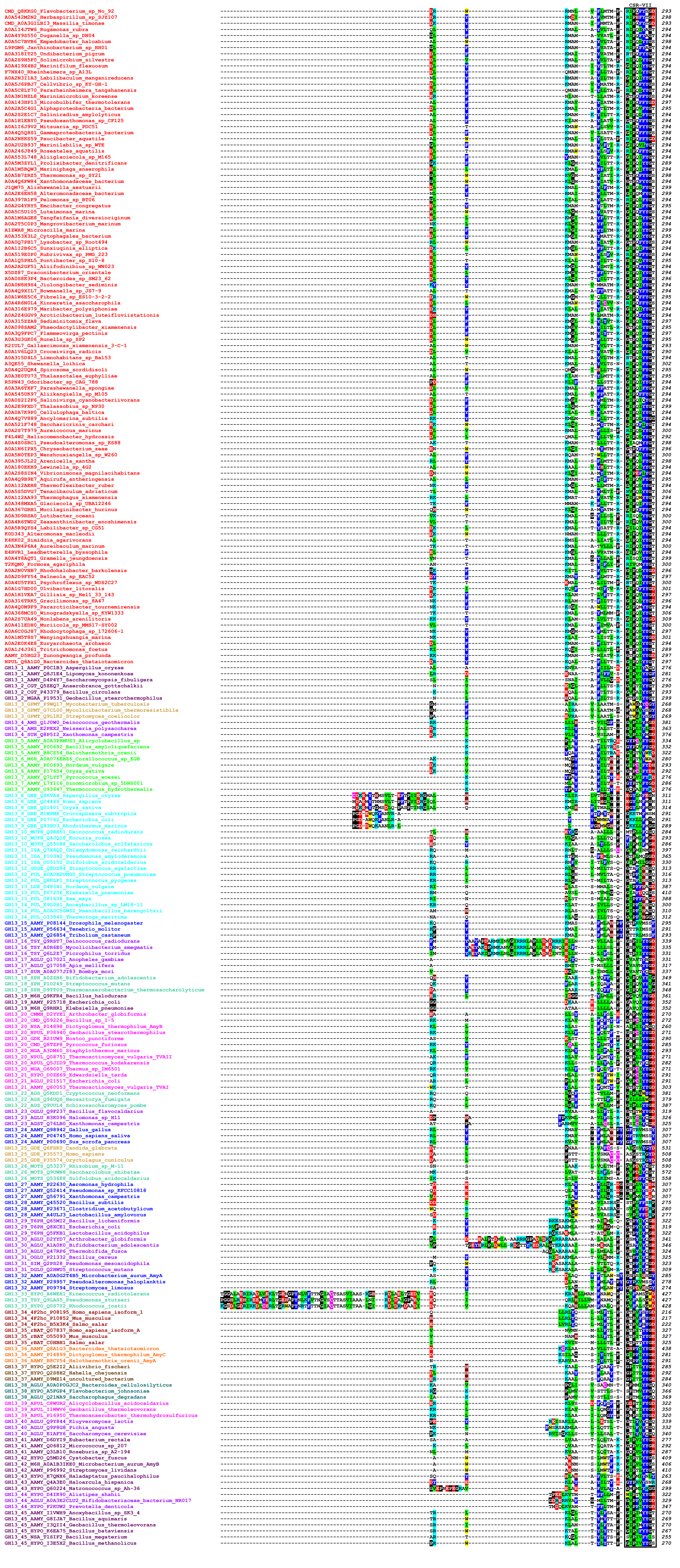
CSR-VII
