# Supplementary material for: A Novel Subfamily GH13_46 of the α-Amylase Family GH13 Represented by the Cyclomaltodextrinase from Flavobacterium sp. No. 92
Source: Molecules. 2022 Dec 9;27(24):8735. doi: 10.3390/molecules27248735 (PMC9781549; doi:10.3390/molecules27248735)
Supplement: Supplementary file 1 [file molecules-27-08735-s001.zip › Supplementary_data_Janecek_Figure_S5_R1.pdf]

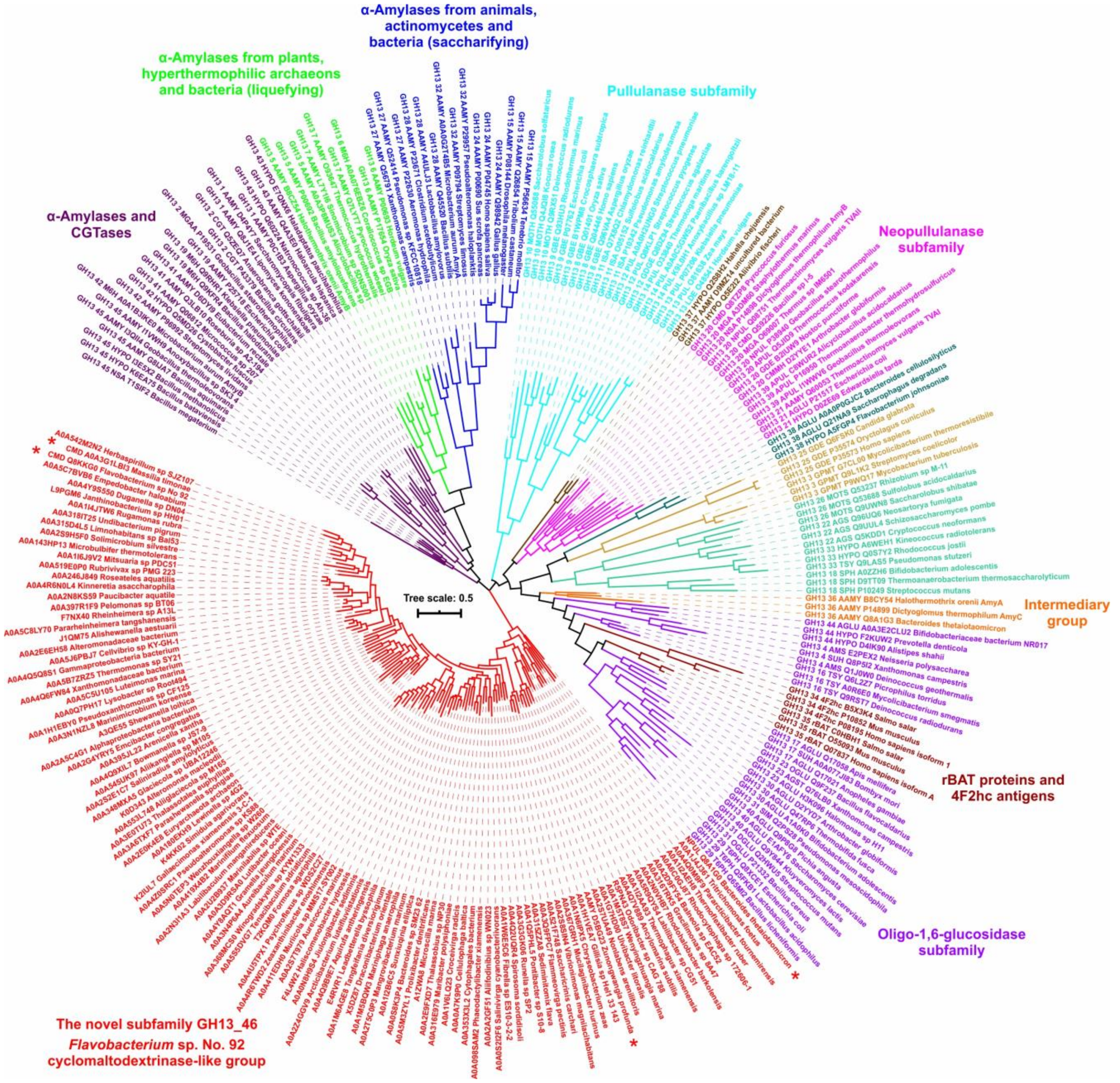

Figure S5. Evolutionary tree of the α-amylase family GH13. The tree covers 253 sequences with focus on the novel subfamily GH13_46 around the cyclomaltodextrinase from *Flavobacterium* sp. No. 92 (for details, see Table S1). The tree is based on the alignment shown in Figure S4, spanning the sequence segment from the beginning of the strand β2 (CSR-VI) to the end of the strand β8 (CSR-VII), i.e. the substantial part of the catalytic TIM-barrel including the domain B. The same but simplified tree without the leaves is presented in Figure 4.
