# Supplementary material for: A Novel Subfamily GH13_46 of the α-Amylase Family GH13 Represented by the Cyclomaltodextrinase from Flavobacterium sp. No. 92
Source: Molecules. 2022 Dec 9;27(24):8735. doi: 10.3390/molecules27248735 (PMC9781549; doi:10.3390/molecules27248735)
Supplement: Supplementary file 1 [file molecules-27-08735-s001.zip › Supplementary_data_Janecek_Table_S1_R1.pdf]

Table S1. List of all 253 sequences of the present study.[a]

No.	Kingdom	Protein[b]	Subfamily	Source	GenBank[c]	UniProt[d]	Length[e]	N-domain[f]
1	Bacteria	CMD	-	*Flavobacterium* sp. No. 92	CAD32957.1	Q8KKG0	619	20-121
2	Bacteria	HYPO	-	*Herbaspirillum* sp. SJZ107	TQK07309.1	A0A542M2N2	634	31-133
3	Bacteria	CMD	-	*Massilia timonae*	AUD56243.1	A0A3G1LBI3	598	1-102
4	Bacteria	HYPO	-	*Rugamonas rubra*	SFL69657.1	A0A1I4JTW6	616	20-121
5	Bacteria	HYPO	-	*Duganella* sp. DN04	TFW16317.1	A0A4Y9S550	618	21-123
6	Bacteria	HYPO	-	*Empedobacter haloabium*	TXE28239.1	A0A5C7BVB6	623	20-124
7	Bacteria	HYPO	-	*Janthinobacterium* sp. HH01	ELX10506.1	L9PGM6	618	22-123
8	Bacteria	HYPO	-	*Undibacterium pigrum*	PXX38649.1	A0A318IT25	618	23-124
9	Bacteria	HYPO	-	*Solimicrobium silvestre*	PRC95188.1	A0A2S9H5F0	619	24-125
10	Bacteria	HYPO	-	*Marinifilum flexuosum*	RKE02552.1	A0A419X4B2	616	21-123
11	Bacteria	HYPO	-	*Rheinheimera* sp. A13L	EGM77553.1	F7NX40	617	20-120
12	Bacteria	HYPO	-	*Labilibaculum manganireducens*	PKQ64047.1	A0A2N3I1A3	616	22-122
13	Bacteria	HYPO	-	*Cellvibrio* sp. KY-GH-1	QEY18047.1	A0A5J6PBJ7	621	26-128
14	Bacteria	HYPO	-	*Pararheinheimera tangshanensis*	TXK82251.1	A0A5C8LY70	617	24-124
15	Bacteria	HYPO	-	*Marinimicrobium koreense*	ROQ21573.1	A0A3N1NZL8	613	20-120
16	Bacteria	HYPO	-	*Microbulbifer thermotolerans*	AMX03475.1	A0A143HP13	616	20-121
17	Bacteria	HYPO	-	*Alphaproteobacteria* bacterium	PCJ38663.1	A0A2A5C4G1	624	28-130
18	Bacteria	HYPO	-	*Saliniradius amylolyticus*	AWL11070.1	A0A2S2E1C7	614	20-121
19	Bacteria	HYPO	-	*Pseudoxanthomonas* sp. CF125	SDQ86321.1	A0A1H1EBY0	630	35-137
20	Bacteria	HYPO	-	*Mitsuaria* sp. PDC51	SFR75721.1	A0A1I6J9V2	650	49-152
21	Bacteria	HYPO	-	*Gammaproteobacteria* bacterium	RYY03769.1	A0A4Q5Q8S1	628	30-132
22	Bacteria	HYPO	-	*Paucibacter aquatile*	PND36270.1	A0A2N8KS59	619	24-127
23	Bacteria	HYPO	-	*Marinilabilia* sp. WTE	PWD99554.1	A0A2U2B937	619	23-125
24	Bacteria	HYPO	-	*Roseateles aquatilis*	OWQ88736.1	A0A246J849	621	20-123
25	Bacteria	HYPO	-	*Aliiglaciecola* sp. M165	TRY32669.1	A0A553L748	618	25-125
26	Bacteria	HYPO	-	*Prolixibacter denitrificans*	GET20604.1	A0A5M3ZYL1	620	28-130
27	Bacteria	HYPO	-	*Mariniphaga anaerophila*	SHF44796.1	A0A1M5BQW3	615	20-122
28	Bacteria	HYPO	-	*Thermomonas* sp. SY21	QDA57577.1	A0A5B7ZRZ5	617	21-121
29	Bacteria	HYPO	-	*Xanthomonadaceae* bacterium	RZA21715.1	A0A4Q6FW84	618	24-126
30	Bacteria	HYPO	-	*Alishewanella aestuarii*	EJI86716.1	J1QM75	628	28-130
31	Bacteria	HYPO	-	*Alteromonadaceae* bacterium	MBJ92230.1	A0A2E6EH58	627	27-129
32	Bacteria	HYPO	-	*Pelomonas* sp. BT06	RIA66095.1	A0A397R1F9	609	21-116
33	Bacteria	HYPO	-	*Emcibacter congregatus*	PHZ85124.1	A0A2G4YRY5	623	28-129

34	Bacteria	HYPO	-	*Luteimonas marina*	TWT19242.1	A0A5C5U105	615	21-119
35	Bacteria	HYPO	-	*Tangfeifania diversioriginum*	SHI35525.1	A0A1M6AGE8	614	20-122
36	Bacteria	HYPO	-	*Mangrovibacterium marinum*	PTN08161.1	A0A2T5C0P3	614	19-121
37	Bacteria	HYPO	-	*Microscilla marina*	EAY25346.1	A1ZWA8	623	23-125
38	Bacteria	HYPO	-	*Cytophagales* bacterium	HBH25349.1	A0A353X3L2	621	24-125
39	Bacteria	HYPO	-	*Lysobacter* sp. Root494	KQY50527.1	A0A0Q7PH17	619	19-121
40	Bacteria	HYPO	-	*Sunxiuqinia elliptica*	SFE51619.1	A0A1I2B6C5	615	20-122
41	Bacteria	HYPO	-	*Rubrivivax* sp. PMG_223	RZI57072.1	A0A519E0P0	621	20-123
42	Bacteria	HYPO	-	*Pontibacter* sp. S10-8	OKL41681.1	A0A1Q5PHL5	616	23-121
43	Bacteria	HYPO	-	*Aliifodinibius* sp. WN023	PAU95624.1	A0A2A2GF51	620	25-127
44	Bacteria	HYPO	-	*Draconibacterium orientale*	AHW59621.1	X5DZ87	612	19-119
45	Bacteria	HYPO	-	*Bacteroides* sp. SM23_62	KPL16521.1	A0A0S8K3P4	617	22-121
46	Bacteria	HYPO	-	*Jiulongibacter sediminis*	KPM48180.1	A0A0N8H9S4	616	22-122
47	Bacteria	HYPO	-	*Bowmanella* sp. JS7-9	TBX20393.1	A0A4Q9XIL7	603	18-112
48	Bacteria	HYPO	-	*Fibrella* sp. ES10-3-2-2	ARK10620.1	A0A1W6E5C6	614	18-120
49	Bacteria	HYPO	-	*Kinneretia asaccharophila*	TDP06663.1	A0A4R6N0L4	630	31-135
50	Bacteria	HYPO	-	*Maribacter polysiphoniae*	PWK25929.1	A0A316E979	618	21-123
51	Bacteria	HYPO	-	*Arcticibacterium luteifluviistationis*	AWW00477.1	A0A2Z4GGV9	609	16-117
52	Bacteria	HYPO	-	*Sediminitomix flava*	PWJ42697.1	A0A315ZZA8	625	26-127
53	Bacteria	HYPO	-	*Phaeodactylibacter xiamenensis*	KGE89165.1	A0A098SAM2	617	20-122
54	Bacteria	HYPO	-	*Flammeovirga pectinis*	AZQ62863.1	A0A3Q9FPC7	623	23-125
55	Bacteria	HYPO	-	*Runella* sp. SP2	AYQ31693.1	A0A3G3GK06	612	21-118
56	Bacteria	HYPO	-	*Gallaecimonas xiamenensis*	EKE74016.1	K2IUL7	608	18-118
57	Bacteria	HYPO	-	*Croceivirga radicis*	OQD42273.1	A0A1V6LQ23	612	20-123
58	Bacteria	HYPO	-	*Limnohabitans* sp. Bal53	PUE38492.1	A0A315D4L5	646	48-149
59	Bacteria	HYPO	-	*Shewanella loihica*	ABO23753.1	A3QE55	654	43-143
60	Bacteria	HYPO	-	*Spirosoma sordidisoli*	RYC70151.1	A0A4Q2UQR4	613	17-119
61	Bacteria	HYPO	-	*Thalassotalea euphylliae*	REL27984.1	A0A3E0TU73	647	51-153
62	Bacteria	HYPO	-	*Odoribacter* sp. CAG:788	CCZ10392.1	R5PN43	624	27-129
63	Bacteria	HYPO	-	*Parashewanella spongiae*	RJY16419.1	A0A3A6TXF7	614	19-119
64	Bacteria	HYPO	-	*Aliikangiella* sp. M105	TQV89873.1	A0A545UK97	597	1-100
65	Bacteria	HYPO	-	*Salinivirga cyanobacteriivorans*	ALO16394.1	A0A0S2I2F6	639	44-146
66	Bacteria	HYPO	-	*Thalassobius* sp. NP30	MBT28616.1	A0A2E9FXD7	644	40-142
67	Bacteria	HYPO	-	*Cellulophaga baltica 18*	AIZ41884.1	A0A0A7K9P0	619	22-124
68	Bacteria	HYPO	-	*Ancylomarina subtilis*	RZT91837.1	A0A4Q7V889	620	24-126
69	Bacteria	HYPO	-	*Saccharicrinis carchari*	SMO91350.1	A0A521F748	615	18-120
70	Bacteria	HYPO	-	*Aureicoccus marinus*	PQJ16480.1	A0A2S7T979	626	25-124
71	Bacteria	HYPO	-	*Haliscomenobacter hydrossis*	AEE54024.1	F4L4W2	630	35-137

72	Bacteria	HYPO	-	*Pseudoalteromonas* sp. KS88	TGE83173.1	A0A4Z0SRC1	618	27-121
73	Bacteria	HYPO	-	*Chryseobacterium zeae*	SIO34056.1	A0A1N6IPX5	618	18-120
74	Bacteria	HYPO	-	*Wenzhouxiangella* sp. W260	KAA9133515.1	A0A5N0TEP3	628	27-128
75	Bacteria	HYPO	-	*Arenicella xantha*	RBP49742.1	A0A395JL22	628	28-130
76	Bacteria	HYPO	-	*Lewinella* sp. 4G2	OAV43703.1	A0A180EKH9	616	25-121
77	Bacteria	HYPO	-	*Vibrionimonas magnilacihabitans*	PQV60703.1	A0A2S8SIN4	615	18-120
78	Bacteria	HYPO	-	*Aquirufa antheringensis*	TBH72092.1	A0A4Q9B9E7	619	20-119
79	Bacteria	HYPO	-	*Thermoflexibacter ruber*	SFE42316.1	A0A1I2AEH8	610	20-118
80	Bacteria	HYPO	-	*Tenacibaculum adriaticum*	TYP99036.1	A0A5S5DVG7	644	35-136
81	Bacteria	HYPO	-	*Thermophagus xiamenensis*	SFE40637.1	A0A1I2AA93	621	25-127
82	Bacteria	HYPO	-	*Glaciecola* sp. UBA12246	HAQ48916.1	A0A348MXA5	635	35-141
83	Bacteria	HYPO	-	*Mucilaginibacter hurinus*	RCH55860.1	A0A367GRH1	616	20-120
84	Bacteria	HYPO	-	*Lutibacter oceani*	REE80026.1	A0A3D9RSA0	622	18-120
85	Bacteria	HYPO	-	*Zeaxanthinibacter enoshimensis*	TDQ33268.1	A0A4R6TWD2	626	25-126
86	Bacteria	HYPO	-	*Labilibacter* sp. CG51	TLX75344.1	A0A5R9QYS4	622	26-128
87	Bacteria	HYPO	-	*Alteromonas macleodii*	AFT78376.1	K0D343	648	41-142
88	Bacteria	HYPO	-	*Simiduia agarivorans*	AFU99326.2	K4KK02	619	20-117
89	Bacteria	HYPO	-	*Aureibaculum marinum*	RPE00171.1	A0A3N4P6A4	625	22-123
90	Bacteria	HYPO	-	*Leadbetterella byssophila*	ADQ17983.1	E4RVR1	604	17-112
91	Bacteria	HYPO	-	*Gramella jeungdoensis*	TEW72194.1	A0A4Y8AQT1	621	20-120
92	Bacteria	HYPO	-	*Formosa agariphila*	CDF80746.1	T2KQM0	635	29-131
93	Bacteria	HYPO	-	*Rhodohalobacter barkolensis*	PKD43577.1	A0A2N0VHB7	624	23-124
94	Bacteria	HYPO	-	*Balneola* sp. EAC52	MAO66358.1	A0A2D9FY54	620	22-123
95	Bacteria	HYPO	-	*Psychroflexus* sp. WDS2C27	TKS55932.1	A0A4U5TPX1	620	18-119
96	Bacteria	HYPO	-	*Ulvibacter litoralis*	SDE98347.1	A0A1G7HD00	617	16-115
97	Bacteria	HYPO	-	*Gillisia* sp. Hel1_33_143	SDS83053.1	A0A1H1VEA7	615	21-118
98	Bacteria	HYPO	-	*Gracilimonas* sp. 8A47	PWN05685.1	A0A316TRK5	627	25-127
99	Bacteria	HYPO	-	*Pararcticibacter tournemirensis*	RXF69831.1	A0A4Q0M9F9	638	42-144
100	Bacteria	HYPO	-	*Winogradskyella* sp. KYW1333	RCT53914.1	A0A368MCS0	648	38-140
101	Bacteria	HYPO	-	*Nonlabens arenilitoris*	PQJ31805.1	A0A2S7UA49	630	15-114
102	Bacteria	HYPO	-	*Muriicola* sp. MMS17-SY002	QBA65661.1	A0A411EDH0	613	6-108
103	Bacteria	HYPO	-	*Rhodocytophaga* sp. 172606-1	QHT67732.1	A0A6C0GJ87	617	20-121
104	Bacteria	HYPO	-	*Wenyingzhuangia marina*	SHH47114.1	A0A1M5T8S7	616	20-116
105	Archaea	HYPO	-	*Euryarchaeota archaeon*	MAS23277.1	A0A2E0K4E8	616	24-125
106	Eucarya	HYPO	-	*Tritrichomonas foetus*	OHS93888.1	A0A1J4J361	615	12-112
107	Bacteria	AAMY	-	*Zunongwangia profunda*	ADF53136.1	D5BG23	615	22-119
108	Bacteria	NPUL	-	*Bacteroides thetaiotaomicron*	AAO78809.1	Q8A1G0	617	23-122

109	Eucarya	AAMY	GH13_1	*Aspergillus oryzae*	CAA31218.1	P0C1B3	499	-
110	Eucarya	AAMY	GH13_1	*Lipomyces kononenkoae*	AAO12212.1	Q8J1E4	499	-
111	Eucarya	AAMY	GH13_1	*Saccharomycopsis fibuligera*	ADD80242.1	D4P4Y7	494	-
112	Bacteria	CGT	GH13_2	*Anaerobranca gottschalkii*	CAH61550.1	Q5ZEQ7	721	-
113	Bacteria	CGT	GH13_2	*Bacillus circulans*	CAA55023.1	P43379	713	-
114	Bacteria	MGAA	GH13_2	*Geobacillus stearothermophilus*	AAA22233.1	P19531	719	-
115	Bacteria	GPMT	GH13_3	*Mycobacterium tuberculosis*	CCP44085.1	P9WQ17	701	-
116	Bacteria	GPMT	GH13_3	*Mycolicibacterium thermoresistibile*	EHI11807.1	G7CL00	696	-
117	Bacteria	GPMT	GH13_3	*Streptomyces coelicolor*	CAB72419.1	Q9L1K2	675	-
118	Bacteria	AMS	GH13_4	*Deinococcus geothermalis*	ABF44874.1	Q1J0W0	650	-
119	Bacteria	AMS	GH13_4	*Neisseria polysaccharea*	EFH23057.1	E2PEX2	636	-
120	Bacteria	SUH	GH13_4	*Xanthomonas campestris*	AAM42629.1	Q8P5I2	637	-
121	Bacteria	AAMY	GH13_5	*Alicyclobacillus* sp. 18711	AWX66236.1	A0A3P8MUS3	514	-
122	Bacteria	AAMY	GH13_5	*Bacillus amyloliquefaciens*	AAA22191.1	P00692	514	-
123	Bacteria	AAMY	GH13_5	*Halothermothrix orenii* (AmyB)	ACL70573.1	B8CZ54	623	-
124	Bacteria	M6H	GH13_6	*Corallococcus sp. EGB*	AII00648.1	A0A076EBZ6	522	-
125	Eucarya	AAMY	GH13_6	*Hordeum vulgare*	AAA32929.1	P00693	438	-
126	Eucarya	AAMY	GH13_6	*Oryza sativa*	AAA33885.1	P17654	434	-
127	Archaea	AAMY	GH13_7	*Pyrococcus woesei*	AAD54338.1	Q7LYT7	460	-
128	Bacteria	AAMY	GH13_7	*Sinomicrobium* sp. 5DNS001	AGD88873.1	L7Y1I6	478	-
129	Archaea	AAMY	GH13_7	*Thermococcus hydrothermalis*	AAC97877.1	O93647	457	-
130	Eucarya	GBE	GH13_8	*Aspergillus oryzae*	BAB69770.1	Q96VA4	689	-
131	Eucarya	GBE	GH13_8	*Homo sapiens*	AAA58642.1	Q04446	702	-
132	Eucarya	GBE	GH13_8	*Oryza sativa*	BAA01584.1	Q01401	820	-
133	Bacteria	GBE	GH13_9	*Crocosphaera subtropica*	ACB51598.1	B1WPM8	773	-
134	Bacteria	GBE	GH13_9	*Escherichia coli*	AAA23872.1	P07762	728	-
135	Bacteria	GBE	GH13_9	*Rhodothermus marinus*	BAB69858.1	Q93HU3	621	-
136	Bacteria	MOTH	GH13_10	*Deinococcus radiodurans*	AAF10042.1	Q9RX51	600	-
137	Bacteria	MOTH	GH13_10	*Kocuria rosea*	AAY83363.1	Q4JQI8	624	-
138	Archaea	MOTH	GH13_10	*Saccharolobus solfataricus*	BAA11010.1	Q55088	559	-
139	Eucarya	ISA	GH13_11	*Chlamydomonas reinhardtii*	AAP85534.1	Q7X8Q2	875	-
140	Bacteria	ISA	GH13_11	*Pseudomonas amyloderamosa*	CAA31754.1	P10342	776	-
141	Archaea	ISA	GH13_11	*Sulfolobus acidocaldarius*	ALU30386.1	O05152	713	-
142	Bacteria	GDGE	GH13_12	*Streptococcus agalactiae*	AAN00098.1	Q8DZ94	1252	-
143	Bacteria	PUL	GH13_12	*Streptococcus pneumoniae*	AAK74446.1	A0A0H2UNG0	1280	-
144	Bacteria	PUL	GH13_12	*Streptococcus pyogenes*	CAD32942.1	Q8KLP1	1165	-
145	Eucarya	LDE	GH13_13	*Hordeum vulgare*	AAD04189.1	O48541	904	-

146	Bacteria	PUL	GH13_13	*Klebsiella pneumoniae*	CAA36431.1	P07206	1090	-
147	Eucarya	PUL	GH13_13	*Zea mays*	AAD11599.1	O81638	962	-
148	Bacteria	PUL	GH13_14	*Anoxybacillus* sp. LM18-11	AEW23439.1	K9L0H1	707	-
149	Bacteria	PUL	GH13_14	*Paenibacillus barengoltzii*	AJP16551.1	A0A0C5GWS2	675	-
150	Bacteria	PUL	GH13_14	*Thermotoga maritima*	CAA04522.1	O33840	843	-
151	Eucarya	AAMY	GH13_15	*Drosophila melanogaster*	AAA92226.1	P08144	494	-
152	Eucarya	AAMY	GH13_15	*Tenebrio molitor*	-	P56634	471	-
153	Eucarya	AAMY	GH13_15	*Tribolium castaneum*	AAA03708.1	Q26854	490	-
154	Bacteria	TSY	GH13_16	*Deinococcus radiodurans*	AAF11586.1	Q9RST7	552	-
155	Bacteria	TSY	GH13_16	*Mycolicibacterium smegmatis*	ABK71531.1	A0R6E0	593	-
156	Archaea	TSY	GH13_16	*Picrophilus torridus*	AAT42654.1	Q6L2Z7	558	-
157	Eucarya	AGLU	GH13_17	*Anopheles gambiae*	CAA60857.1	Q17021	498	-
158	Eucarya	AGLU	GH13_17	*Apis mellifera*	BAA11466.1	Q17058	567	-
159	Eucarya	SUH	GH13_17	*Bombyx mori*	BAP18683.1	A0A077JI83	606	-
160	Bacteria	SPH	GH13_18	*Bifidobacterium adolescentis*	AAO33821.1	A0ZZH6	504	-
161	Bacteria	SPH	GH13_18	*Streptococcus mutans*	CAA30846.1	P10249	481	-
162	Bacteria	SPH	GH13_18	*Thermoanaerobacterium thermosaccharolyticum*	ADL69407.1	D9TT09	488	-
163	Bacteria	M6H	GH13_19	*Bacillus halodurans*	BAB04132.1	Q9KFR4	958	-
164	Bacteria	AAMY	GH13_19	*Escherichia coli*	CAA41740.1	P25718	676	-
165	Bacteria	M6H	GH13_19	*Klebsiella pneumoniae*	BAA88434.1	Q9RHR1	677	-
166	Bacteria	CMMH	GH13_20	*Arthrobacter globiformis*	BAI67607.1	D2YYE1	450	-
167	Bacteria	CMD	GH13_20	*Bacillus* sp. I-5	AAA92925.1	Q59226	558	-
168	Bacteria	NSA	GH13_20	*Dictyoglomus thermophilum* (AmyB)	CAA31586.1	P14898	562	-
169	Bacteria	NPUL	GH13_20	*Geobacillus stearothermophilus*	AAA22622.1	P38940	588	-
170	Bacteria	GDE	GH13_20	*Nostoc punctiforme*	ACC84362.1	B2IUW9	488	-
171	Archaea	CMD	GH13_20	*Pyrococcus furiosus*	AAL82063.1	Q8TZP8	645	-
172	Archaea	MGA	GH13_20	*Staphylothermus marinus*	ABN69720.1	A3DM60	696	-
173	Bacteria	NPUL	GH13_20	*Thermoactinomyces vulgaris* (TVAII)	BAA02473.1	Q08751	585	-
174	Archaea	APUL	GH13_20	*Thermococcus kodakarensis*	BAD85166.1	Q5JID9	765	-
175	Bacteria	MGA	GH13_20	*Thermus sp. IM6501*	AAC15072.1	O69007	588	-
176	Bacteria	HYPO	GH13_21	*Edwardsiella tarda*	ACY83800.1	D0ZE69	596	-
177	Bacteria	AGLU	GH13_21	*Escherichia coli*	CAA42498.1	P21517	604	-
178	Bacteria	AAMY	GH13_21	*Thermoactinomyces vulgaris* (TVAI)	BAA02471.1	Q60053	666	-
179	Eucarya	AGS	GH13_22	*Cryptococcus neoformans*	AAW44814.1	Q5KDD1	2430	-
180	Eucarya	AGS	GH13_22	*Neosartorya fumigata*	AAL28129.1	Q96UQ6	2420	-
181	Eucarya	AGS	GH13_22	*Schizosaccharomyces pombe*	BAA76558.1	Q9UUL4	2352	-
182	Bacteria	OGLU	GH13_23	*Bacillus flavocaldarius*	BAB18518.1	Q9F237	529	-
183	Bacteria	AGLU	GH13_23	*Halomonas* sp. H11	BAL49684.1	H3K096	538	-

184	Bacteria	ATGS	GH13_23	*Xanthomonas campestris*	BAC87873.1	H3K096	538	-
185	Eucarya	AAMY	GH13_24	*Gallus gallus*	AAC60246.1	Q98942	512	-
186	Eucarya	AAMY	GH13_24	*Homo sapiens* (saliva)	AAH63129.1	P04745	511	-
187	Eucarya	AAMY	GH13_24	*Sus scrofa* (pancreas)	AAF02828.1	P00690	511	-
188	Eucarya	GDE	GH13_25	*Candida glabrata*	CAG59721.1	Q6FSK0	1528	-
189	Eucarya	GDE	GH13_25	*Homo sapiens*	AAB41040.1	P35573	1532	-
190	Eucarya	GDE	GH13_25	*Oryctolagus cuniculus*	AAA16364.1	P35574	1555	-
191	Bacteria	MOTS	GH13_26	*Rhizobium* sp. M-11	BAA11186.1	Q53237	772	-
192	Archaea	MOTS	GH13_26	*Saccharolobus shibatae*	AAF17554.1	Q9UWN8	728	-
193	Archaea	MOTS	GH13_26	*Sulfolobus acidocaldarius*	ALU30387.1	Q53688	720	-
194	Bacteria	AAMY	GH13_27	*Aeromonas hydrophila*	AAA21936.1	P22630	464	-
195	Bacteria	AAMY	GH13_27	*Pseudomonas* sp. KFCC10818	AAA86836.1	Q52414	466	-
196	Bacteria	AAMY	GH13_27	*Xanthomonas campestris*	AAA27591.1	Q56791	475	-
197	Bacteria	AAMY	GH13_28	*Bacillus subtilis*	CAA30643.1	Q45520	477	-
198	Bacteria	AAMY	GH13_28	*Clostridium acetobutylicum*	AAA63759.2	P23671	760	-
199	Bacteria	AAMY	GH13_28	*Lactobacillus amylovorus*	ABO77965.1	A4ULJ3	478	-
200	Bacteria	T6PH	GH13_29	*Bacillus licheniformis*	AAU22384.1	Q65MI2	562	-
201	Bacteria	T6PH	GH13_29	*Escherichia coli*	BAB38639.1	Q8XCE1	551	-
202	Bacteria	T6PH	GH13_29	*Lactobacillus acidophilus*	AAV42863.1	Q5FKB1	554	-
203	Bacteria	AGLU	GH13_30	*Arthrobacter globiformis*	BAI67603.1	D2YYD7	567	-
204	Bacteria	AGLU	GH13_30	*Bifidobacterium adolescentis*	BAF39233.1	A1A0K0	590	-
205	Bacteria	AGLU	GH13_30	*Thermobifida fusca*	AAZ54871.1	Q47RP6	544	-
206	Bacteria	OGLU	GH13_31	*Bacillus cereus*	CAA37583.1	P21332	558	-
207	Bacteria	SIM	GH13_31	*Pseudomonas mesoacidophila*	ABC33903.1	Q2PS28	584	-
208	Bacteria	DGLU	GH13_31	*Streptococcus mutans*	BAE79634.1	Q2HWU5	531	-
209	Bacteria	AAMY	GH13_32	*Microbacterium aurum* (AmyA)	AKG25402.1	A0A0G2T4B5	1417	-
210	Bacteria	AAMY	GH13_32	*Pseudoalteromonas haloplanktis*	CAA41481.1	P29957	668	-
211	Bacteria	AAMY	GH13_32	*Streptomyces limosus*	AAA88554.1	P09794	566	-
212	Bacteria	HYPO	GH13_33	*Kineococcus radiotolerans*	ABS05210.1	A6WEH1	774	-
213	Bacteria	TSY	GH13_33	*Pseudomonas stutzeri*	AAF26837.1	Q9LAS5	689	-
214	Bacteria	HYPO	GH13_33	*Rhodococcus jostii*	ABG96354.1	Q0S7Y2	814	-
215	Eucarya	4F2hc	GH13_34	*Homo sapiens* (isoform 1)	AAA35489.1	P08195	630	-
216	Eucarya	4F2hc	GH13_34	*Mus musculus*	AAA35489.1	P10852	526	-
217	Eucarya	4F2hc	GH13_34	*Salmo salar*	ACI33885.1	B5X3K4	510	-
218	Eucarya	rBAT	GH13_35	*Homo sapiens* (isoform A)	AAA35500.1	Q07837	685	-
219	Eucarya	rBAT	GH13_35	*Mus musculus*	BAA24427.1	O55093	685	-
220	Eucarya	rBAT	GH13_35	*Salmo salar*	ACN11390.1	C0HBH1	681	-
221	Bacteria	AAMY	GH13_36	*Bacteroides thetaiotaomicron*	AAB42174.1	Q8A1G3	692	-

222	Bacteria	AAMY	GH13_36	*Dictyoglomus thermophilum* (AmyC)	CAA34072.1	P14899	499	-
223	Bacteria	AAMY	GH13_36	*Halothermothrix orenii* (AmyA)	ACL70223.1	B8CY54	515	-
224	Bacteria	HYPO	GH13_37	*Aliivibrio fischeri*	AAW86764.1	Q5E2I2	634	-
225	Bacteria	HYPO	GH13_37	*Hahella chejuensis*	ABC33052.1	Q2S8H2	556	-
226	Bacteria	AAMY	GH13_37	Uncultured bacterium	ADK21254.1	D9MZ14	638	-
227	Bacteria	AGLU	GH13_38	*Bacteroides cellulosilyticus*	ALJ62728.1	A0A0P0GJC2	565	-
228	Bacteria	HYPO	GH13_38	*Flavobacterium johnsoniae*	ABQ05620.1	A5FGP4	610	-
229	Bacteria	AGLU	GH13_38	*Saccharophagus degradans*	ABD79820.1	Q21NA9	624	-
230	Bacteria	APUL	GH13_39	*Alicyclobacillus acidocaldarius*	ACV59878.1	C8WUR2	1299	-
231	Bacteria	APUL	GH13_39	*Geobacillus thermoleovorans*	AFI70750.1	I1WWV6	1655	-
232	Bacteria	APUL	GH13_39	*Thermoanaerobacter thermohydrosulfuricus*	AAA23205.1	P16950	1475	-
233	Eucarya	AGLU	GH13_40	*Kluyveromyces lactis*	CAB46746.1	Q9Y844	583	-
234	Eucarya	AGLU	GH13_40	*Pichia angusta*	AAF69018.2	Q9P8G8	564	-
235	Eucarya	AGLU	GH13_40	*Saccharomyces cerevisiae*	ADK27710.1	E1AFY6	589	-
236	Bacteria	AAMY	GH13_41	*Eubacterium rectale*	CBK91127.1	D6DYI9	1364	-
237	Bacteria	AAMY	GH13_41	*Micrococcus* sp. 207	CAA39321.1	Q06812	1104	-
238	Bacteria	AAMY	GH13_41	*Roseburia* sp. A2-194	CAJ20070.1	Q3LB10	1674	-
239	Bacteria	HYPO	GH13_42	*Cystobacter fuscus*	AAW03335.1	Q5MD26	978	-
240	Bacteria	M6H	GH13_42	*Microbacterium aurum* (AmyB)	AOF40721.1	A0A1B3IKE0	1278	-
241	Bacteria	AAMY	GH13_42	*Streptomyces lividans*	CAB06816.1	P96992	993	-
242	Archaea	HYPO	GH13_43	*Haladaptatus paucihalophilus*	EFW93629.1	E7QNX6	404	-
243	Archaea	AAMY	GH13_43	*Haloarcula hispanica*	CAI64586.1	Q4A3E0	433	-
244	Archaea	M3H	GH13_43	*Natronococcus sp. Ah-36*	BAA05516.1	Q60224	504	-
245	Bacteria	HYPO	GH13_44	*Alistipes shahii*	CBK63352.1	D4IK90	559	-
246	Bacteria	AGLU	GH13_44	Bifidobacteriaceae bacterium	RFT33048.1	A0A3E2CLU2	566	-
247	Bacteria	HYPO	GH13_44	*Prevotella denticola*	AEA20197.1	F2KUW2	581	-
248	Bacteria	AAMY	GH13_45	*Anoxybacillus* sp. SK3_4	JF932307.1	I1VWH9	505	-
249	Bacteria	AAMY	GH13_45	*Bacillus aquimaris* (BaqA)	AER68125.1	G8IJA7	512	-
250	Bacteria	AAMY	GH13_45	*Geobacillus thermoleovorans*	AFK08971.1	I3QII4	513	-
251	Bacteria	HYPO	GH13_45	*Bacillus bataviensis*	EKN70296.1	K6EA75	508	-
252	Bacteria	NSA	GH13_45	*Bacillus megaterium* (BmaN1)	AGT45938.1	T1SIF2	504	-
253	Bacteria	HYPO	GH13_45	*Bacillus methanolicus*	EIJ81893.1	I3E5X2	511	-

[a] The first 108 sequences represent the newly proposed subfamily GH13_46 represented by the cyclomaltodextrinase from *Flavobacterium* sp. No. 92 [24]; the remaining 109-253 sequences have been selected to represent the 45 GH13 subfamilies – 3 members from each subfamily, except for the subfamilies GH13_20 (10 members) and GH13_45 (6 members).. The colours used for highlighting the sequences from individual subfamilies reflect their clustering in the evolutionary tree (Fig. 4 and Fig. S5).

[b] The individual enzymes are abbreviated as follows: CMD, cyclomaltodextrinase; HYPO, hypothetical enzyme; AAMY, α-amylase; NPUL, neopullulanase; CGT, cyclodextrin glucanotransferase; MGAA, maltogenic α-amylase; GPMT, α-1,4-glucan: phosphate α-maltosyltransferase; AMS, amylosucrase; SUH, sucrose hydrolase; M6H, maltohexaose-

producing amylase; GBE, α-glucan branching enzyme; MOTH, maltooligosyltrehalose trehalohydrolase; ISA, isoamylase; GDGE, glycogen-degrading enzyme; PUL, pullulanase; LDE, limit dextrinase; TSY, trehalose synthase; AGLU, α-glucosidase; SPH, sucrose phosphorylase; CMMH, cyclic maltosyl-maltose hydrolase; NSA, non-specified amylase; MGA, maltogenic amylase; APUL, amylopullulanase; AGS, α-1,3-glucan synthase; OGLU, oligo-1,6-glucosidase; ATGS, α-transglucosidase; GDE, glycogen debranching enzyme; MOTS, maltooligosyltrehalose trehalosynthase; T6PH, trehalose-6-phosphate hydrolase; SIM, sucrose isomerase; DGLU, dextran glucosidase; 4F2hc, 4F2 heavy-chain antigen; rBAT, amino acid transport protein rBAT; M3H, maltotriose-producing amylase.

[c,d] The accession numbers from the GenBank and UniProt databases, respectively.

[e] The length of the enzyme (i.e. number of amino acid residues).

[f] The position of the N-terminal domain in sequences of the newly proposed subfamily around the cyclomaltodextrinase from *Flavobacterium* sp. No. 92 (proteins Nos 1-108).
