# Supplementary material for: A Novel Subfamily GH13_46 of the α-Amylase Family GH13 Represented by the Cyclomaltodextrinase from Flavobacterium sp. No. 92
Source: Molecules. 2022 Dec 9;27(24):8735. doi: 10.3390/molecules27248735 (PMC9781549; doi:10.3390/molecules27248735)
Supplement: Supplementary file 1 [file molecules-27-08735-s001.zip › Supplementary_data_Janecek_Table_S3_R1.pdf]

Table S2. Tertiary structure comparison of the full-length cyclomaltodextrinase from *Flavobacterium* sp. No. 92 with representatives of individual GH13 subfamilies.[a]

Subfamily	Enzyme[b]	Source	PDB[c]	Cα	RMSD
GH13_1	AAMY	*Aspergillus oryzae*	2TAA	348	1.75
GH13_2	CGT	*Bacillus circulans*	1CDG	375	1.48
GH13_3	GPMT	*Mycobacterium tuberculosis*	4U33	330	1.70
GH13_4	AMS	*Deinococcus geothermalis*	3UCQ	342	1.71
GH13_5	AAMY	*Bacillus amyloliquefaciens*	3BH4	272	1.77
GH13_6	AAMY	*Hordeum vulgare*	1HT6	275	1.81
GH13_7	AAMY	*Pyrococcus woesei*	3QGV	280	1.72
GH13_8	GBE	*Homo sapiens*	4BZY	320	1.69
GH13_9	GBE	*Cyanothece* sp. ATCC 51142	5GQU	342	1.62
GH13_10	MOTH	*Deinococcus radiodurans*	2BHU	319	1.61
GH13_11	ISA	*Sulfolobus solfataricus*	2VNC	322	1.72
GH13_12	PUL	*Streptococcus pneumoniae*	2YA0	328	1.62
GH13_13	LDE	*Hordeum vulgare*	4J3S	325	1.73
GH13_14	PUL	*Anoxybacillus* sp. LM18-11	3WDH	317	1.76
GH13_15	AAMY	*Tenebrio molitor*	1CLV	271	1.79
GH13_16	TSY	*Mycolicibacterium smegmatis*	3ZO9	342	1.58
GH13_17	SUH	*Bombyx mori*	6LGA	347	1.64
GH13_18	SPH	*Bifidobacterium adolscentis*	1R7A	304	1.72
GH13_19	M6H	*Bacillus halodurans*	model (1TCM)	366	1.51
GH13_20	CMMH	*Arthrobacter globiformis*	5ZXG	361	1.47
GH13_20	CMD	*Bacillus* sp. I-5	1EA9	370	1.50
GH13_20	NPUL	*Geobacillus stearothermophilus*	1JOH	365	1.48
GH13_20	GDE	*Nostoc punctiforme*	2WC7	342	1.42
GH13_20	CMD	*Pyrococcus furiosus*	4AEF	336	1.37
GH13_20	MGA	*Staphylothermus marinus*	4AEE	349	1.53
GH13_20	NPUL	*Thermoactinomyces vulgaris* (TVAII)	1BVZ	363	1.65
GH13_20	PUL	*Thermococcus_kodakarensis*	5OT1	364	1.46
GH13_20	MGA	*Thermus* sp. IM6501	1GVI	365	1.52
GH13_21	AAMY	*Thermoactinomyces vulgaris* (TVAI)	1UH3	354	1.45
GH13_22	AGS	*Cryptococcus neoformans*	model (1CYG)	372	1.48
GH13_23	ATGS	*Xanthomonas campestris*	6AAV	340	1.61
GH13_24	AAMY	*Homo sapiens*	1HNY	246	1.82
GH13_25	GDE	*Candida grablata*	5D06	283	1.90
GH13_26	MOTS	*Sacharolobus shibatae*	5ZCR	305	1.64
GH13_27	AAMY	*Aeromonas hydrophila*	model (6M4K)	242	1.85
GH13_28	AAMY	*Bacillus subtilis*	1BAG	254	1.72
GH13_29	T6PH	*Bacillus licheniformis*	5BRP	308	1.73
GH13_30	AGLU	*Arthrobacter globiformis*	model (3ZOA)	332	1.56
GH13_31	OGLU	*Bacillus cereus*	1UOK	324	1.66
GH13_32	AAMY	*Pseudoalteromonas haloplanktis*	1AQH	240	1.68
GH13_33	TSY	*Pseudomonas stutzeri*	model (6LI9)	327	1.69
GH13_34	4F2hc	*Homo sapiens*	2DH2	290	1.66
GH13_35	rBAT	*Homo sapiens*	6LI9	342	1.74
GH13_36	AAMY	*Bacteroides thetaiotaomicron*	3K8K	336	1.52
GH13_37	AAMY	uncultured bacterium	5H05	343	1.60
GH13_38	AGLU	*Bacteroides cellulosilitycus*	model (1JIB)	353	1.50
GH13_39	APUL	*Alicyclobacillus acidolcalarius*	model (2D0G)	355	1.45

GH13_40	AGLU	*Sacharomyces cerevisiae*	3A47	308	1.83
GH13_41	AAMY	*Eubacterium rectale*	model (2YA1)	310	1.64
GH13_42	M6H	*Microbacterium aurum*	model (1QHO)	362	1.50
GH13_43	AAMY	*Haloarcula hispanica*	model (1TCM)	311	1.53
GH13_44	AGLU	Bifidobacteriaceae bacterium	model (3ZOA)	331	1.56
GH13_45	AAMY	*Geobacillus thermoleovorans*	4E2O	381	1.52
GH13_45	NSA	*Bacillus megaterium*	model (4E2O)	364	1.54

[a] Results of superposition of the full-length cyclomaltodextrinase from *Flavobacterium* sp. No. 92 ([24]; PDB code: 1H3G) with representatives from individual GH13 subfamilies are shown as the number of corresponding Cα atoms and the RMSD value in Å (the last two columns).

[b] The individual enzymes are abbreviated as follows: AAMY, α-amylase; CGT, cyclodextrin glucanotransferase; GPMT, α-1,4-glucan: phosphate α-maltosyltransferase; AMS, amylosucrase; GBE, α-glucan branching enzyme; MOTH, maltooligosyltrehalose trehalohydrolase; ISA, isoamylase; PUL, pullulanase; LDE, limit dextrinase; TSY, trehalose synthase; SUH, sucrose hydrolase; SPH, sucrose phosphorylase; M6H, maltohexaose-producing amylase; CMMH, cyclic maltosyl-maltose hydrolase; CMD, cyclomaltodextrinase; NPUL, neopullulanase; GDE, glycogen debranching enzyme; MGA, maltogenic amylase; AGS, α-1,3-glucan synthase; ATGS, α-transglucosidase; MOTS, maltooligosyltrehalose trehalosynthase; T6PH, trehalose-6-phosphate hydrolase; AGLU, α-glucosidase; OGLU, oligo-1,6-glucosidase; 4F2hc, 4F2 heavy-chain antigen; rBAT, amino acid transport protein rBAT; APUL, amylopullulanase; NSA, non-specified amylase.

[c] PDB codes from the Protein Data Bank (if available); for models, the PDB code of a template PDB is shown in parenthesis.
