# Supplementary material for: A Novel Subfamily GH13_46 of the α-Amylase Family GH13 Represented by the Cyclomaltodextrinase from Flavobacterium sp. No. 92
Source: Molecules. 2022 Dec 9;27(24):8735. doi: 10.3390/molecules27248735 (PMC9781549; doi:10.3390/molecules27248735)
Supplement: Supplementary file 1 [file molecules-27-08735-s001.zip › Supplementary_data_Janecek_Table_S4_R1.pdf]

Table S3. Tertiary structure comparison of the N-terminal domain of cyclomaltodextrinase from *Flavobacterium* sp. No. 92 with individual representatives of real SBD CBM families.[a]

CBM family	Source[b]	PDB code (UniProt)[c]	Cα	RMSD (Å)
CBM20	GH15 GAMY, *Aspergillus niger*	1AC0	45	1.99
CBM21	GH15 GAMY, *Rhizopus oryzae*	2V8M	53	2.05
CBM25	GH13_19 M6H, *Bacillus halodurans*	2C3W	44	2.04
CBM26	GH13_19 M6H, *Bacillus halodurans*	2C3H	38	1.51
CBM34	GH13_21 AAMY, *Thermoactinomyces vulgaris* (TVA-I)	1UH4	48	1.74
CBM41	GH13_14 PUL, *Thermotoga maritima*	2J73	35	1.97
CBM45 (model)	AGWD, *Solanum tuberosum*	Template: 2C3W (Q9AWA5)	38	2.09
CBM48	AMPK β1-subunit, *Rattus norvegicus*	1Z0M	40	1.81
CBM53 (model)	GT5 SS3, *Arabidopsis thaliana*	Template: 2C3W (F4IAG2)	41	2.04
CBM58	GH13_36 AAMY, *Bacteroides thetaiotaomicron* (SusG)	3K8M	39	1.89
CBM68	GH13_14 PUL *Anoxybacillus* sp.	3WDJ	39	1.94
CBM69	GH13_37 AAMY uncultured bacterium	5X5S	33	2.03
CBM82 (model)	GH13_41 AAMY *Eubacterium rectale*	Template: 2C3H (D6DYI9)	30	2.05
CBM83 (model)	GH13_41 AAMY *Eubacterium rectale*	Template: 2J73 (D6DYI9)	38	1.94
N-domain[d]	GH13_5 AAMY, *Halothermothrix orenii* (AmyB)	3BC9	62	1.77

[a] Results of superposition of the *Flavobacterium* sp. No. 92 cyclomaltodextrinase N-terminal domain ([24]; PDB code: 1H3G) with its SBD CBM counterparts are shown as the number of corresponding Cα atoms and the RMSD value (the last two columns). For those with no three-dimensional structure available until now (CBM45, CBM53, CBM82 and CBM83), a homology model has been created based on a template.

[b] The individual enzymes are abbreviated as follows: GAMY, glucoamylase; M6H, maltohexaose-producing amylase; AAMY, α-amylase; PUL, pullulanase; AGWD, α-glucan, water dikinase; AMPK, AMP-activated protein kinase; SS3, starch synthase 3.

[c] UniProt means the accession No. from the UniProt database for the sequence of the enzyme possessing the particular CBM for which the three-dimensional structure has not been solved until now.

[d] Although the domain N from the GH13_5 α-amylase AmyB from *H. orenii* [43] has not been assigned any SBD CBM family as yet [14], it is shown for comparison here since it exhibits a significant structural similarity with the N-terminal domain of the studied cyclomaltodextrinase.
